# Autophagy- and oxidative stress-related protein deregulation mediated by extracellular vesicles of human MJD/SCA3 iPSC-derived neuroepithelial stem cells and differentiated neural cultures

**DOI:** 10.1038/s41419-025-07659-0

**Published:** 2025-05-15

**Authors:** Liliana S. Mendonça, Ricardo Moreira, Daniel Henriques, Mónica Zuzarte, Teresa M. Ribeiro-Rodrigues, Henrique Girão, Luís Pereira de Almeida

**Affiliations:** 1 Center for Neurosciences and Cell Biology, University of Coimbra, Coimbra, Portugal; 2 Center for Innovative Biomedicine and Biotechnology, University of Coimbra, Coimbra, Portugal; 3 Institute of Interdisciplinary Research, University of Coimbra, Coimbra, Portugal; 4 Faculty of Pharmacy of the University of Coimbra, Coimbra, Portugal; 5 Faculty of Medicine, Coimbra Institute for Clinical and Biomedical Research (iCBR), University of Coimbra, Coimbra, Portugal; 6 Clinical Academic Center of Coimbra (CACC), Coimbra, Portugal

**Keywords:** Molecular neuroscience, Mechanisms of disease, Multipotent stem cells

## Abstract

Extracellular vesicles (EVs) have been associated with the transport of molecules related to the pathological processes in neurodegenerative diseases. Machado-Joseph disease (MJD) is a neurodegenerative disorder triggered by mutant ataxin-3 protein that causes protein misfolding and aggregation resulting in neuronal death. To evaluate EVs’ role in the potential spread of disease-associated factors in MJD, in this study, EVs were isolated from human Control (CNT) and MJD induced-pluripotent stem cell-derived neuroepithelial stem cells (iPSC-derived NESC) and their differentiated neural cultures (cell cultures composed of neurons and glia). EVs were characterized and investigated for their ability to interfere with cell mechanisms known to be impaired in MJD. The presence of mRNA and proteins related to autophagy, cell survival, and oxidative stress pathways, and the mutant ataxin-3, was evaluated in the EVs. SOD1, p62, and Beclin-1 were found present both in CNT and MJD EVs. Lower levels of the p62 autophagy-related protein and higher levels of the oxidative stress-related SOD1 protein were found in MJD EVs. The oxidative stress-related *CYCS* mRNA and autophagy-related *SQSTM1*, *BECN1*, *UBC*, *ATG12*, and *LC3B* mRNAs were detected in EVs and no significant differences in their levels were observed between CNT and MJD EVs. The internalization of EVs by human CNT neurons was demonstrated, and no effect of the EVs administration was observed on cell viability. Moreover, the incubation of MJD EVs (isolated from NESC or differentiated neural cultures) with human CNT differentiated neural cells resulted in the reduction of SOD1 and autophagy-related proteins ATG3, ATG7, Beclin-1, LC3B, and p62 levels. Finally, a tendency for accumulation of ataxin-3-positive aggregates in CNT differentiated neural cells co-cultured with MJD differentiated neural cells was observed. Overall, our data indicate that EVs carry autophagy- and oxidative stress-related proteins and mRNAs and provide evidence of MJD EVs-mediated interference with autophagy and oxidative stress pathways.

## Introduction

Machado-Joseph disease (MJD), also known as spinocerebellar ataxia type 3 (SCA3), is a severe neurodegenerative disorder exhibiting extensive neuronal death affecting diverse brain regions [1], such as the cerebellum [2, 3]. MJD is caused by a mutation in the *ATXN3* gene, which is translated in an expanded polyglutamine (polyQ) tract in the ataxin-3 protein. This polyQ expansion causes protein misfolding and aggregation [4, 5] and mutant ataxin-3 protein accumulates in neuronal intranuclear inclusions [5, 6]. We and others previously reported that proteins associated with autophagy, such as Beclin-1, p62, and LC3B, are entrapped within mutant ataxin-3 aggregates [7], which causes the autophagy impairments observed, given that these proteins will not be available to carry out their functions. Furthermore, downregulation of mRNAs encoding autophagy-associated proteins has also been found [8] and activation of autophagy mediates strong alleviation of neuropathology in animal models [7, 9].

Extracellular vesicles (EVs) are cell membrane-derived vesicles secreted by cells [10] able to transport in their lumen cargoes such as nucleic acids and proteins [11, 12]. Their cargo profile is highly dependent on the cellular context [10, 13] and it has been shown that these vesicles can carry molecules associated with pathological processes, including disease-spreading cues [10].

Despite the large evidence of pathological spreading in several neurodegenerative diseases, the available data regarding disease-spreading cues in MJD and the potential role of EVs in this process is scarce [10]. Elucidation of the potential contribution of EVs in MJD pathology spreading and progression might provide alternative therapeutic strategies and new biomarkers for use in clinical trials [14–16].

Accordingly, the main goal of this work was to assess the role of EVs in MJD neuropathology, specifically their role in autophagy and oxidative stress, and the mutant ataxin-3 spreading between diseased and healthy cells. EVs obtained from human CNT and MJD iPSC-derived NESC (CNT and MJD NESC-EVs) and from their differentiated neural cultures (CNT and MJD Neural-EVs) were isolated and characterized for physical parameters (size and particle number), EVs’ specific markers, protein and RNA content, internalization by human neurons, and impact on cell viability. The effect on p62, LC3B, Beclin-1, ATG7, ATG3, and SOD1 protein levels after the administration of CNT and MJD NESC-EVs and Neural-EVs to the differentiated neural cultures was evaluated. Mutant ataxin-3 spreading from MJD to CNT cells was evaluated in an indirect co-culture model. Altogether, our data provide evidence that MJD EVs when internalized by healthy neuronal cultures spread MJD-associated mechanisms, namely by promoting a reduction in the levels of proteins related to autophagy and oxidative stress.

## Results

### Physical characterization of MJD and CNT EVs isolated from the culture media of human iPSC-derived NESC and their differentiated neural cultures

**Fig. 1:**
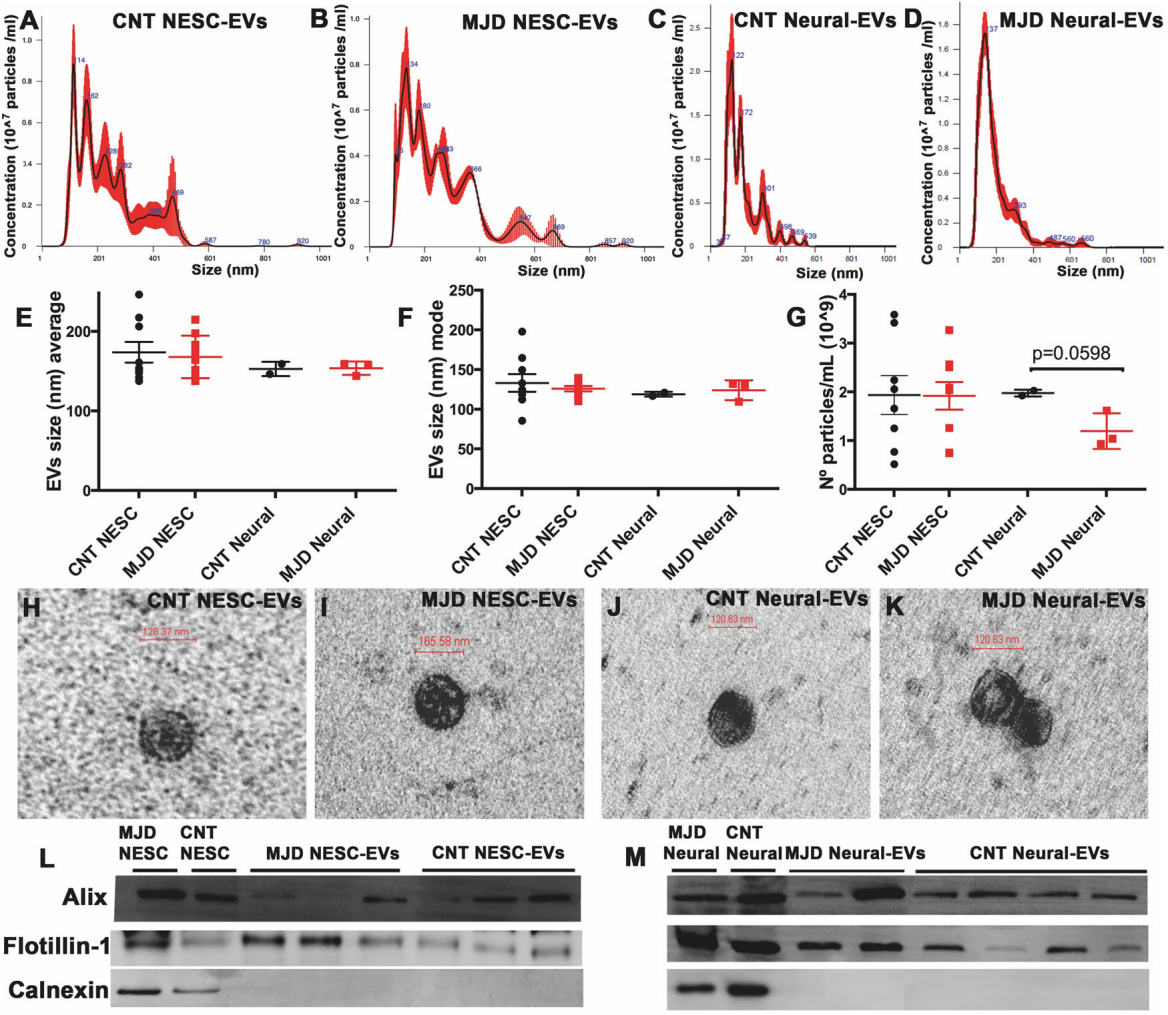
Characterization of MJD and CNT EVs obtained from human iPSC-derived NESC and their differentiated neural cell cultures. EVs isolated from the culture media of **A**, **B**, **E**–**I**, **L** iPSC-derived NESC (NESC-EVs) and **C**, **D**, **E**–**G**, **J**, **K**, **M** neural cell cultures differentiated from iPSC-derived NESC (Neural-EVs) were characterized for their physical properties and the expression of positive and negative EVs’ markers. NESC-EVs from MJD and CNT cells were characterized for their **A**, **B** size distribution, **E** size average, **F** size mode, and **G** number of particles per mL with Nanoparticle Tracking Analysis (NTA). Size and morphology of **H** CNT and **I** MJD NESC-EVs evaluated by Transmission Electron Microscopy (TEM). **L** Western blot representative image showing Alix and Flotillin-1 (positive protein markers) and no Calnexin (negative marker) expression in CNT (CNT NESC-EVs) and MJD (MJD NESC-EVs) EVs, as compared with the cells of origin (MJD NESC and CNT NESC), *n* = 9. Neural-EVs from MJD and CNT cells were also compared for their **C**, **D** size distribution, **E** size average, **F** size mode, and **G** number of particles per mL with NTA. **E**–**G** Data are expressed as mean ± SEM, unpaired t-test with Welch’s correction; NESC-EVs *n* = 7–8 and Neural-EVs *n* = 3. Size and morphology of **J** CNT and **K** MJD Neural-EVs analyzed by TEM. **M** Representative Western blot image showing that both CNT and MJD Neural-EVs expressed Alix and Flotillin-1 and no Calnexin was detected, as compared with the expression of these proteins in the cells of origin (MJD Neural and CNT Neural), *n* = 5–6.

EVs were isolated by the differential centrifugation method from the culture media of human MJD and CNT iPSC-derived NESC (NESC-EVs) and neural cell cultures composed of glia and neurons obtained through differentiation of human iPSC-derived NESC (Neural-EVs). The obtained EVs were then subjected to characterization (Fig. 1, Fig. S1), namely particle size distribution, average size, and concentration were evaluated through Nanoparticle Tracking Analysis (NTA). For NESC-EVs, no significant differences were observed between MJD and CNT EVs regarding size distribution (Fig. 1A, B, E, F), average size (Fig. 1E), and mode size (Fig. 1F). An average size of 173.69 ± 13.05 nm was observed for CNT NESC-EVs and 167.83 ± 8.92 nm for MJD NESC-EVs (Fig. 1A, B), which are values in accordance with the EVs’ size reported by other authors [14]. As for differentiated neural cell cultures-derived EVs (Neural-EVs), no significant differences were also observed between CNT and MJD EVs regarding size distribution (Fig. 1C–F), mean average size (Fig. 1E), and mode size (Fig. 1F). The average size of CNT Neural-EVs was 152.72 ± 6.33 nm and of MJD Neural-EVs was 153.72 ± 4.84 nm (Fig. 1E). Interestingly, a tendency for higher heterogeneity in the size distribution of NESC-EVs as compared with the Neural-EVs was observed (Fig. 1A–D), namely in the D10, D50, and D90 percentile that indicate the size below which 10%, 50%, and 90%, respectively, of all particles are found (Fig. S1). Regarding EVs’ concentration, no difference was observed between CNT and MJD NESC-EVs. CNT NESC-EVs concentration was 1.95 ± 0.40 × 10^9^ particles/ml and MJD NESC-EVs was 1.92 ± 0.28 × 10^9^ particles/ml (Fig. 1G). Regarding the particle concentration of Neural-EVs, CNT Neural-EVs was 1.98 ± 0.05 × 10^9^ particles/ml while MJD Neural-EVs was 1.19 ± 0.21 × 10^9^ particles/ml. Thus, a tendency of 1.66 times lower particle concentration was observed for the MJD Neural-EVs (Fig. 1G). Transmission Electron Microscopy (TEM) was used to evaluate the structure and membrane integrity of CNT and MJD NESC-EVs (Fig. 1H, I), and CNT and MJD Neural-EVs (Fig. 1J, K). Round structures with sizes between 100 and 200 nm were observed for all the analyzed EVs, which is in accordance with the NTA observations. Finally, as expected, western blot analysis showed that CNT and MJD NESC-EVs (Fig. 1L) and Neural-EVs (Fig. 1M) have proteins commonly enriched in EVs, such as ALIX and Flotillin-1, and the negative marker Calnexin was absent in the EV samples as compared to the cells from which the EVs were isolated (CNT and MJD NESC and differentiated neural cultures). Therefore, the obtained EVs samples meet the current standards regarding EVs characterization.

### Evaluation of protein and mRNA cargo in EVs

**Fig. 2:**
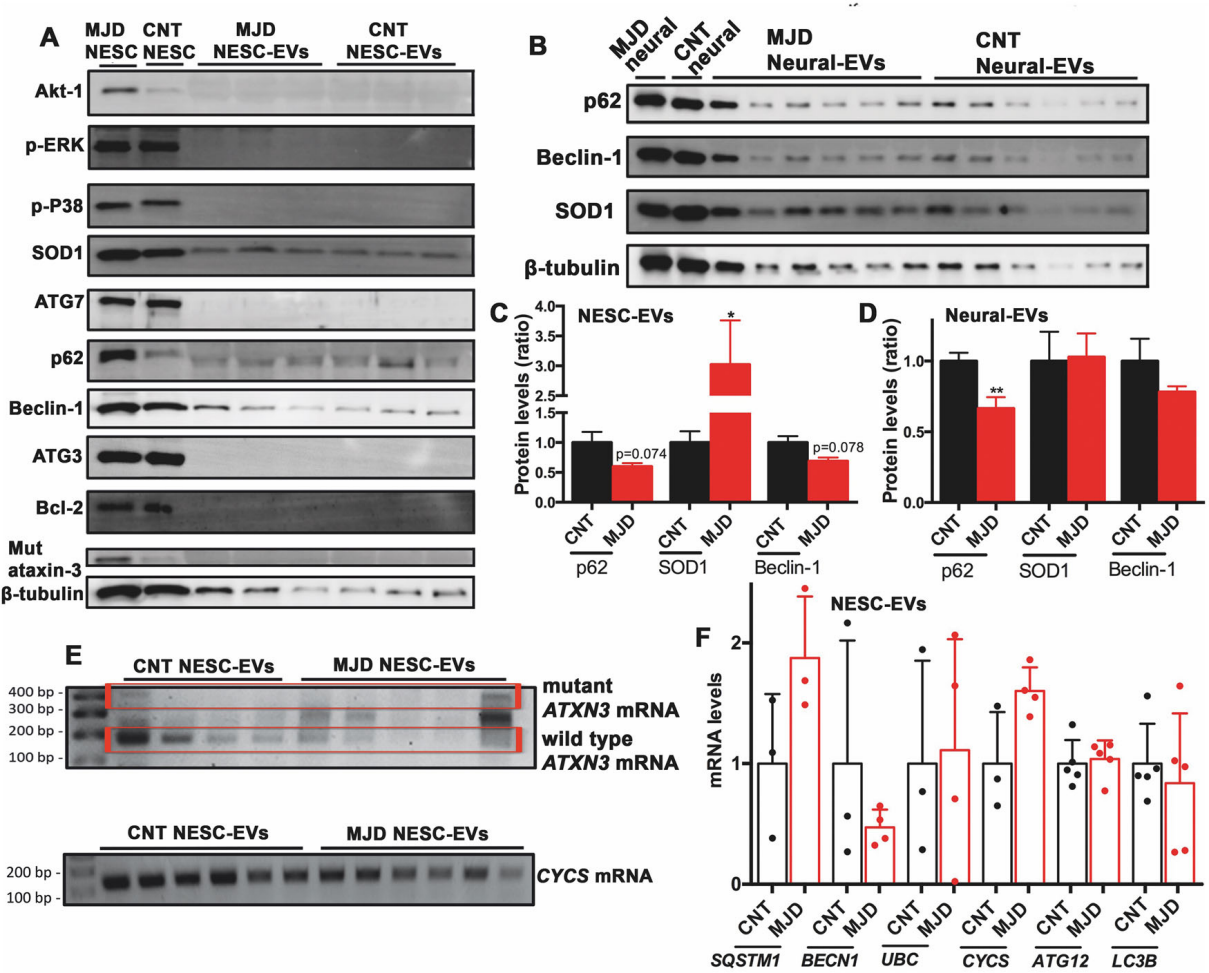
Characterization of protein and RNA cargo in CNT and MJD EVs. **A** The presence of a set of proteins related to autophagy (ATG7, ATG3, p62, and Beclin-1), cell survival (Akt-1, p-ERK, Bcl2, and p-P38), oxidative stress (SOD1), mutant ataxin-3 (Mut ataxin-3), and the β-tubulin was evaluated by Western blot in CNT and MJD NESC-EVs and the respective cells of origin (MJD NESC and CNT NESC). The proteins p62, Beclin-1, and SOD1 were detected in CNT and MJD NESC-EVs and were further evaluated in **B** CNT and MJD Neural-EVs. The p62, Beclin-1, and SOD1 protein levels were quantified in **C** NESC-EVs and **D** Neural-EVs by western blot and normalized for β-tubulin and CNT EVs levels. **C** p62 *n* = 8, Beclin-1 *n* = 3, SOD-1 *n* = 9; **D** *n* = 6. **E** Evaluation of mutant and wild-type *ATXN3* and CYCS mRNA presence in CNT and MJD NESC-EVs by semi-quantitative RT-PCR; *n* = 4–6. **F** Quantification of *SQSTM1*, *BECN1*, *UBC*, *CYCS*, *ATG12*, and *LC3B* mRNA levels in CNT and MJD NESC-EVs through qRT-PCR normalized for GAPDH and CNT mRNA levels; *n* = 4–5. Data are expressed as mean ± SEM, *p < 0.05, **p < 0.01, unpaired t-test with Welch’s correction.

The presence of mRNA and proteins related to autophagy, cell survival, and oxidative stress pathways, described as deregulated in the MJD context, and the disease-causing mutant ataxin-3, was evaluated in the CNT and MJD EVs (Fig. 2). The presence of cell survival-related proteins Akt-1, Bcl-2, p-ERK, and p-P38, the autophagy-related proteins ATG3, ATG7, p62, and Beclin-1, the free radical scavenging SOD1 protein, and the mutant ataxin-3 protein were screened in the CNT and MJD NESC-EVs by western blot analysis (Fig. 2A). From the screened proteins, Akt-1, Bcl-2, p-ERK, p-P38, ATG3, ATG7, and mutant ataxin-3 were not detected, while p62, Beclin-1, and SOD1 were found present both in CNT and MJD NESC-EVs. Furthermore, these proteins were quantified to assess possible differences in expression between the two populations of EVs (Fig. 2B, C). Beclin-1 and p62 proteins tended to decrease in MJD NESC-EVs (Fig. 2C). Whereas SOD1 protein levels were 3 times (3.021 ± 0.739) increased in MJD NESC-EVs compared to CNT NESC-EVs. Considering Neural-EVs, p62, Beclin-1, and SOD1 proteins were also screened and quantified by western blot (Fig. 2B, D). No significant differences in SOD1 and Beclin-1 protein levels were detected between CNT and MJD Neural-EVs. Whereas p62 protein levels were found 34% (0.664 ± 0.081) significantly decreased in MJD Neural-EVs (Fig. 2D). These data indicate that both NESC-EVs and Neural-EVs carry autophagy- and oxidative stress-related proteins that might impact cell pathways upon internalization. Moreover, CNT and MJD EVs present different expression of p62 and SOD1 protein levels.

Regarding mRNA cargo detection and quantification, through semi-quantitative RT-PCR, the presence of wild-type *ATXN3* mRNA (182 bp) was detected in both CNT and MJD NESC-EVs, whereas mutant *ATXN3* (353 bp) was not robustly detected. A band appeared with the same size as the mutant *ATXN3* mRNA in just 1 of the 5 MJD NESC-EVs samples (Fig. 2E). The presence of Cytochrome C (CYCS – related with oxidative stress) mRNA was also detected in CNT and MJD EVs. Through RT-qPCR the autophagy-related *SQSTM1*, *BECN1*, *UBC*, *ATG12*, and *LC3B* mRNA were detected and quantified as well as the oxidative stress-related *CYCS* mRNA levels in CNT and MJD NESC-EVs (Fig. 2F). No significant differences in the mRNA levels were detected between CNT and MJD EVs, although a high variability in the mRNA levels was observed across the different samples.

### Cellular internalization of EVs and their impact on oxidative stress and autophagy of human neuronal cultures

**Fig. 3:**
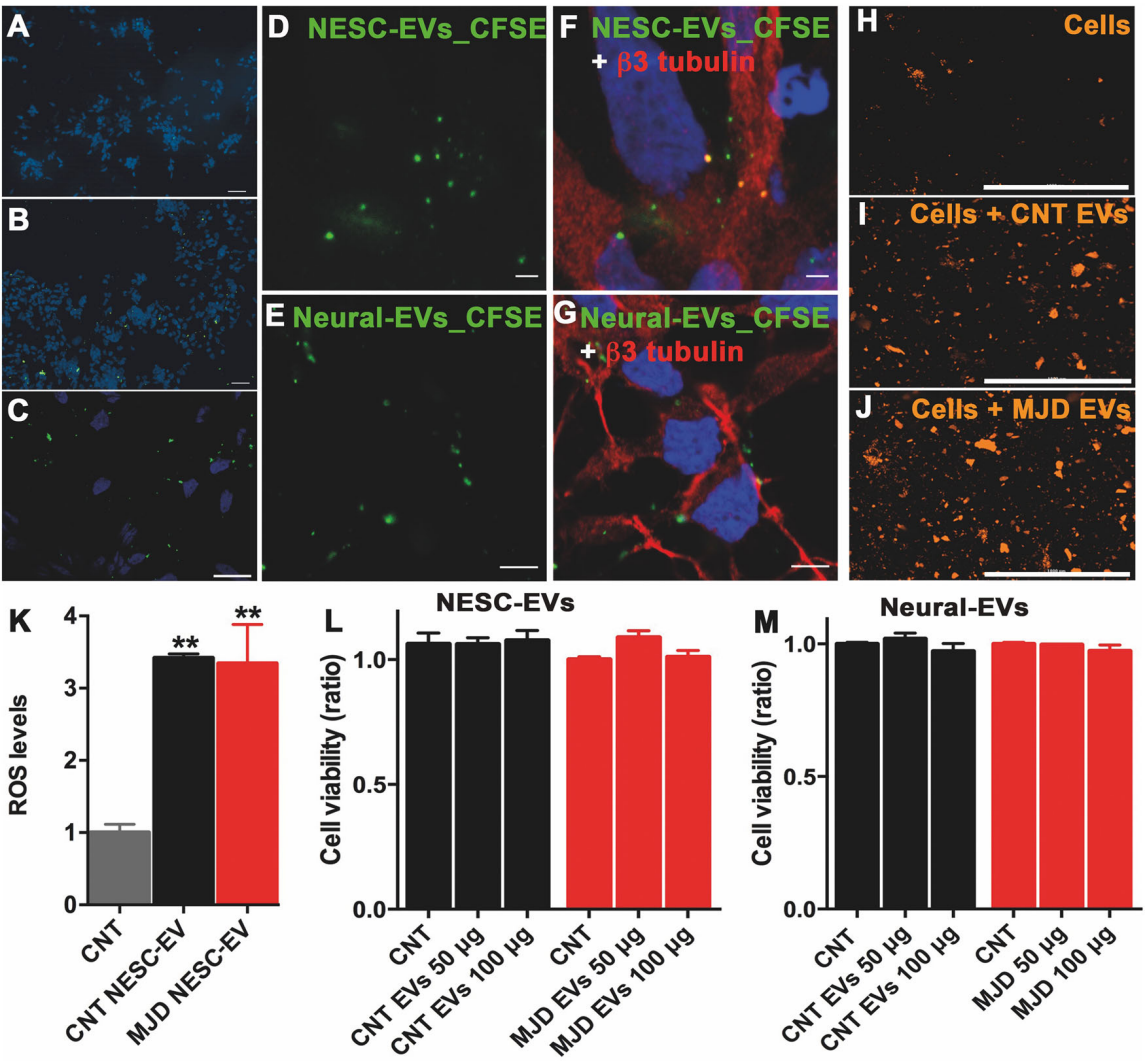
EVs’ cell internalization, reactive oxygen species (ROS) production stimulation, and effect on cell viability. EVs isolated from the culture medium of human iPSC-derived NESC (NESC-EVs) and from neural cell cultures differentiated from iPSC-derived NESC (Neural-EVs) were incubated with human CNT differentiated neural cell cultures. **A**–**G** Representative confocal fluorescence microscopy images showing CFSE (green)-labeled EVs internalization by the human differentiated neural cells, DAPI: blue, n = 3. **A** Cells incubated with the negative CNT (PBS + CFSE) presented no CFSE labeling, while cells incubated with the CFSE-labeled **B**, **D**–**F** NESC-EVs and **C**, **E**–**G** Neural-EVs showed the green-labeled EVs, namely in **F**, **G** neurons stained for β3 tubulin (red), scale bars: A, B: 50 μm; C: 20 μm; D, F: 2 μm, and E, G: 5 μm. **H**–**K** Reactive oxygen species (ROS) levels assessment in the human differentiated neural cell cultures after incubation with NESC-EVs. Representative fluorescence image of ROS (orange) levels in differentiated neural cell cultures (Cells) **H** before and after incubation for 1 h with 100 μg/ml of **I** CNT (Cells + CNT EVs) and **J** MJD (Cells + MJD EVs) NESC-EVs. **K** ROS levels in differentiated neural cell cultures without treatment (CNT cells, CNT) and treated with CNT (CNT NESC-EVs) and MJD (MJD NESC-EVs) NESC-EVs were assessed by the relative fluorescence units (RFU) quantification of ROS fluorescence probe normalized for the CNT; *n* = 6. **L**, **M** Viability of differentiated neural cell cultures incubated for 3 days with 50 and 100 μg/ml (50 and 100 μg) of **L** CNT and MJD NESC-EVs and **M** CNT and MJD Neural-EVs normalized for CNT (not treated differentiated neural cell cultures); *n* = 4–12. Data are expressed as mean ± SEM, One-way ANOVA with Tukey’s multiple comparison test, **p < 0.01.

Before evaluating the effect of EVs administration on cell autophagy and oxidative stress, the EVs’ internalization by human neurons was assessed (Fig. 3A–G). For this, NESC-EVs (Fig. 3B, D, F) and Neural-EVs (Fig. 3C, E, G) were labeled with carboxyfluorescein diacetate succinimidyl ester (CFSE) and incubated with human differentiated neural cultures for 14 h. Cells incubated with the negative CNT (CFSE + PBS) (Fig. 3A) exhibited no green fluorescence indicating that no free CFSE probe is available and that the observed fluorescence in confocal microscopy pictures (Fig. 3B–G) is from CFSE-labeled EVs. Thus, data demonstrated that CFSE-labeled EVs (green puncta) were internalized by the human neurons (β3 tubulin-positive cells, red) indicating that the isolation and storage process does not render the EVs unable to enter the cells.

Reactive oxygen species (ROS) are cell activity by-products and their overproduction has been associated with several human diseases including neurodegenerative diseases [17]. As SOD1, an important ROS processing enzyme, was found in the EVs, the effect of the EVs administration on cellular ROS production was evaluated (Fig. 3H–K). As expected, ROS production was detected in CNT differentiated neural cultures (Fig. 3H). Moreover, upon 100 μg/ml of CNT (Fig. 3I, K) and MJD (Fig. 3J, K) NESC-EVs administration, a 3.42 ± 0.11 and 3.34 ± 0.54 times increase, respectively, in ROS production was observed, and no significant differences were observed between cells treated with CNT and MJD EVs.

Given that ROS are associated with cell toxicity [17], it was also evaluated the impact of NESC-EVs (Fig. 3L) and Neural-EVs (Fig. 3M) administration in the viability of human neuronal cultures. Thus, 3 days after the incubation with 50 and 100 μg/ml of CNT and MJD EVs, the cell viability was evaluated by the resazurin reduction assay, and no cell toxicity was observed.

**Fig. 4:**
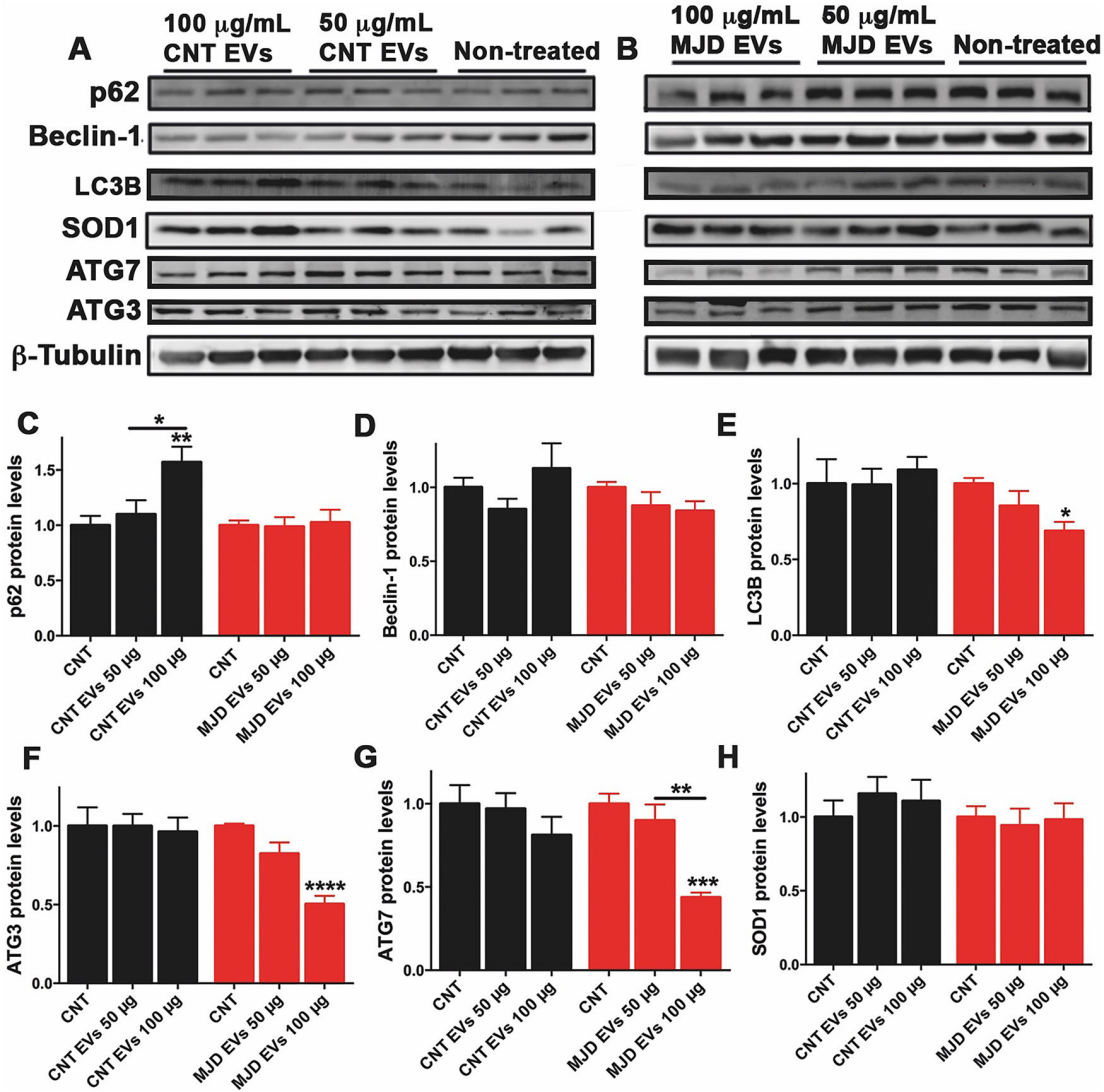
MJD NESC-EVs downregulate the levels of proteins related to autophagy and oxidative stress. **A**–**H** Western blot analysis of proteins related to autophagy (p62, Beclin-1, LC3B, ATG3, and ATG7) and oxidative stress (SOD1) in differentiated neural cell cultures after 3 days incubation with 50 and 100 μg/ml of CNT (CNT EVs) and MJD (MJD EVs) NESC-derived EVs (NESC-EVs) compared with non-treated cells (CNT, non-treated). Representative Western blot images of p62, Beclin-1, LC3B, ATG3, ATG7, SOD1, and β-Tubulin protein levels after incubation with **A** CNT and **B** MJD NESC-EVs. Quantification of **C** p62, **D** Beclin-1, **E** LC3B, **F** ATG3, **G** ATG7, and **H** SOD1 protein levels in human differentiated neural cell cultures incubated with 50 and 100 μg/ml (50 and 100 μg) of CNT (CNT EVs) and MJD (MJD EVs) NESC-EVs, normalized for β-Tubulin and non-treated cells (CNT). Data are expressed as mean ± SEM; *n* = 9 for CNT EVs and *n* = 11 for MJD EVs. Data are expressed as mean ± SEM, *p < 0.05, **p < 0.01, ***p < 0.001, ****p < 0.0001, One-way ANOVA with Tukey’s multiple comparison test.

CNT and MJD NESC-EVs, at 50 and 100 μg/ml, were incubated with differentiated neural cultures. Three days after the EVs incubation, the cell expression of SOD1 and autophagy-related proteins p62, Beclin-1, LC3B, ATG3, and ATG7 was evaluated by western blot (Fig. 4). Data demonstrated a 1.569 ± 0.142-fold increase in the p62 protein levels in cells treated with 100 μg/ml of CNT NESC-EVs (Fig. 4C), whereas no change in p62 levels was detected upon MJD EVs treatment. No significant difference in the Beclin-1 expression was detected either with 50 or 100 μg/ml of CNT and MJD EVs (Fig. 4D). Regarding LC3B protein levels, a 31.20% decrease (0.688 ± 0.059) was observed in cells treated with 100 μg/ml of MJD EVs (Fig. 4E), while CNT EVs promoted no change in LC3B protein levels. Additionally, a 50% decrease in ATG3 (0.504 ± 0.052, Fig. 4F) and a 56.20% decrease in ATG7 (0.438 ± 0.028, Fig. 4G) protein levels in cells incubated with 100 μg/ml of MJD EVs were observed, while CNT EVs promoted no changes in these protein levels. Finally, no significant differences in SOD1 protein levels were detected in cells treated with either CNT or MJD EVs. Additionally, the p62, Beclin-1, LC3B, ATG3, ATG7, and SOD1 protein levels were also evaluated 2 weeks after cell incubation with CNT and MJD NESC-EVs (Fig. S2). Data revealed that most previously detected changes in protein levels were no longer present. Interestingly, SOD1 protein levels that were not changed after 3 days of incubation, at 2 weeks post administration of 100 μg/ml of MJD EVs (Fig. S2H) exhibited a 50% reduction (0.505 ± 0.096).

**Fig. 5:**
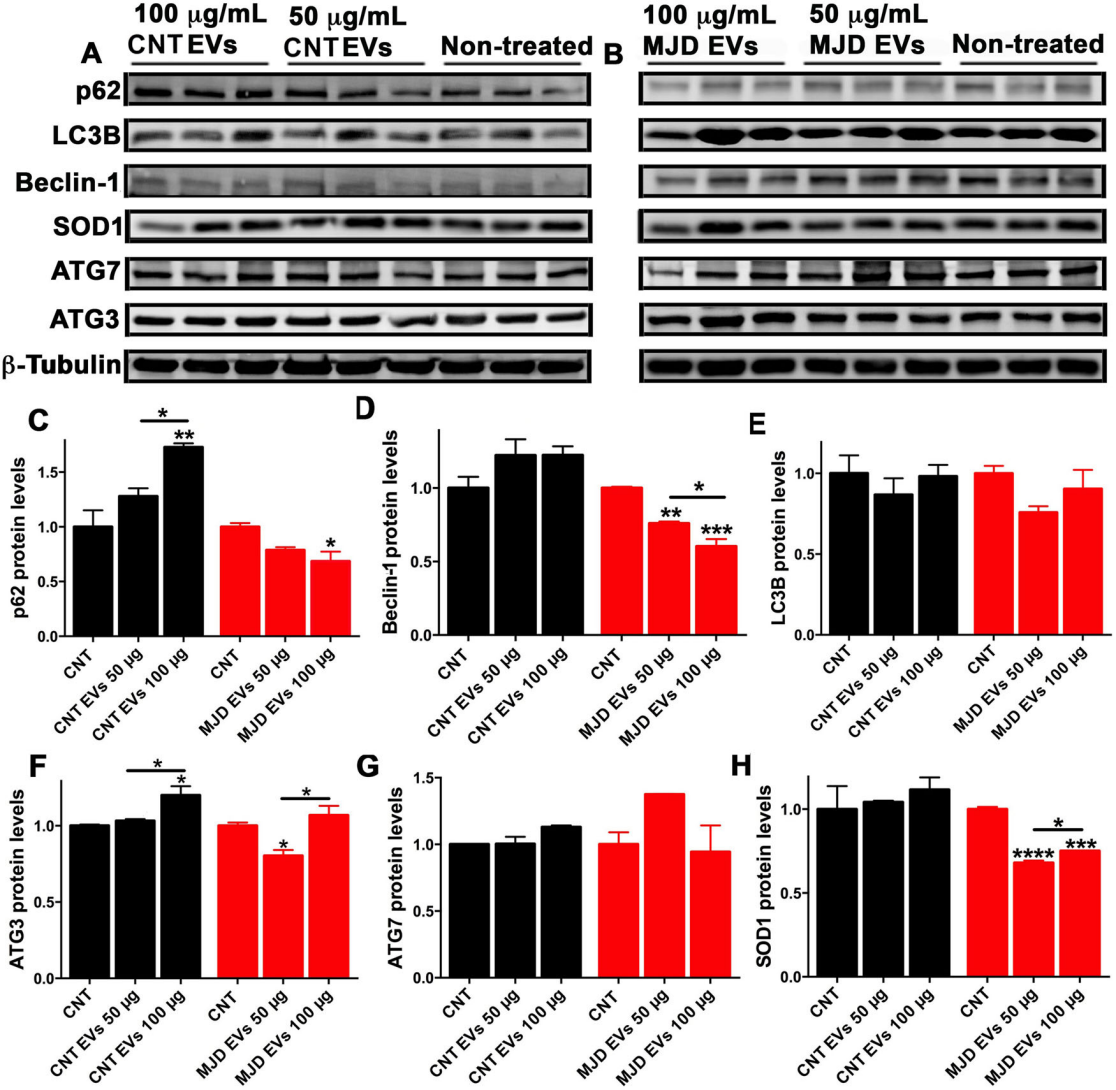
MJD Neural-EVs downregulate levels of proteins related to autophagy and oxidative stress. **A**–**H** Western blot analysis of proteins related to autophagy (p62, Beclin-1, LC3B, ATG3, and ATG7) and oxidative stress (SOD1) in differentiated neural cell cultures after 3 days incubation with 50 and 100 μg/ml of CNT (CNT EVs) and MJD (MJD EVs) differentiated neural cell cultures-derived EVs (Neural-EVs) compared with non-treated cells (CNT, non-treated). Representative Western blot images of p62, Beclin-1, LC3B, ATG3, ATG7, SOD1, and β-Tubulin protein levels after incubation with **A** CNT and **B** MJD Neural-EVs. Quantification of **C** p62, **D** Beclin-1, **E** LC3B, **F** ATG3, **G** ATG7, and **H** SOD1 protein levels in human differentiated neural cell cultures incubated with 50 and 100 μg/ml (50 and 100 μg) of CNT (CNT EVs) and MJD (MJD EVs) Neural-EVs, normalized for β-Tubulin and non-treated cells (CNT); *n* = 3. Data are expressed as mean ± SEM, *p < 0.05, **p < 0.01, ***p < 0.001, ****p < 0.0001, One-way ANOVA with Tukey’s multiple comparison test.

Furthermore, CNT and MJD Neural-EVs were also incubated with differentiated neural cell cultures, and 3 days later, the cell expression of SOD1, p62, Beclin-1, LC3B, ATG3, and ATG7 protein levels was evaluated by western blot (Fig. 5). A significant increase of over 70% (1.73 ± 0.036) in the p62 protein levels was observed for cells treated with 100 μg/ml of CNT Neural-EVs (Fig. 5C), which corroborates the observed p62 levels enhancement promoted by CNT NESC-EVs. Interestingly, cells treated with 100 μg/ml of MJD Neural-EVs exhibited a significant 31.60% reduction (0.68 ± 0.089) in the p62 protein levels. These results indicate that CNT and MJD Neural-EVs trigger opposite effects on p62 protein levels of recipient cells. Regarding Beclin-1 protein levels (Fig. 5D), CNT Neural-EVs promoted no change in its levels, while a reduction of 24.10% (0.759 ± 0.013) and 40% (0.603 ± 0.050) was observed in cells treated with 50 μg/ml and 100 μg/ml of MJD Neural-EVs, respectively. No difference in the LC3B expression was observed for cells treated with either CNT or MJD Neural-EVs (Fig. 5E). Moreover, ATG3 protein levels were found to be increased by 20% (1.20 ± 0.059) in cells incubated with 100 μg/ml CNT Neural-EVs (Fig. 5F), while a 20% reduction (0.803 ± 0.039) was detected upon treatment with 50 μg/ml MJD Neural-EVs. Regarding the ATG7 expression, no significant changes were observed between cells treated with CNT and MJD Neural-EVs (Fig. 5G). Finally, CNT Neural-EVs promoted no changes in SOD1 levels, while a 32.10% (0.679 ± 0.014) and 25% (0.75 ± 0.003) reduction was detected with 50 and 100 μg/ml of MJD Neural-EVs treatment (Fig. 5H). Altogether, these results clearly indicate that proteins related to autophagy were enhanced upon treatment with CNT EVs and decreased with MJD EVs. Additionally, MJD EVs promoted a reduction in the SOD1 protein levels. These data revealed a potential protective role triggered by CNT EVs, while the EVs obtained from MJD patient cells downregulated the autophagy-related and antioxidant protein levels.

### Evaluation of mutant ataxin-3 spreading from MJD to CNT cells in human differentiated neural cultures

**Fig. 6:**
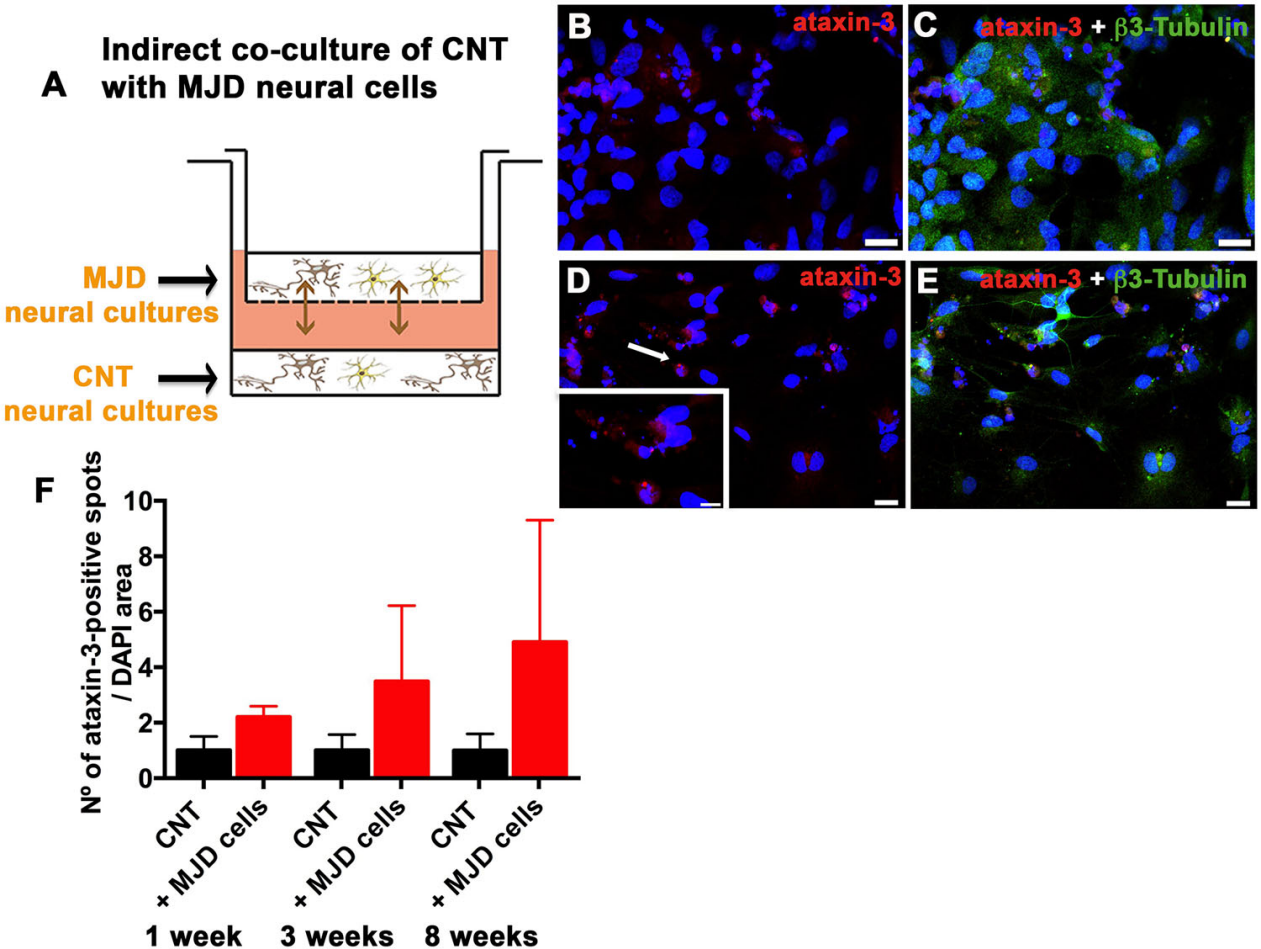
Mutant ataxin-3 spreading from MJD to CNT differentiated neural cells in an indirect co-culture system. **A** Illustration of the in vitro model used to assess mutant ataxin-3 spreading from MJD to CNT differentiated neural cells through the indirect co-culture of CNT cells (in the bottom of the well) with MJD differentiated neural cells in an insert (at the top of the well). **B**–**E** Representative immunofluorescence confocal microscopy images of **B**, **C** CNT differentiated neural cell cultures grown alone and **D**, **E** indirectly co-cultured for 8 weeks with MJD differentiated neural cell cultures, collected at this time point, and stained for the presence of mutant ataxin-3 aggregates (red); DAPI (blue), Scale bars: 10 μm. Lower insert: higher magnification image of ataxin-3-positive spots/aggregates (red) in DAPI-positive cell nucleus (blue). **F** Quantification of ataxin-3-positive spots in the cell nucleus (DAPI) of CNT cells (CNT) and CNT cells co-cultured with MJD cells (CNT + MJD cells) for 1, 3, and 8 weeks, collected at the end of the co-culture time, and stained for the presence of mutant ataxin-3 aggregates; *n* = 3, 2, and 2 independent experiments for 1, 3, and 8 weeks of cells’ co-culturing, respectively. Data are expressed as mean ± SEM, unpaired t-test with Welch’s correction.

To uncover whether there is spreading of the mutant ataxin-3 protein from MJD to CNT cells, the presence of mutant ataxin-3 inclusions was evaluated in CNT differentiated neural cells growing in indirect contact with MJD differentiated neural cells (Fig. 6). MJD and CNT neural cell cultures composed of neurons and glia differentiated from human iPSC-derived NESC were co-cultured. Human MJD cells were plated in the transwell inserts sharing culture media with the CNT cells through a membrane with 1 μm pore (Fig. 6A) for 1, 3, and 8 weeks. Immediately after this co-culture time points, cells were collected and fluorescence confocal microscopy images of ataxin-3 (red) immunolabeling revealed the presence of more pronounced red puncta, indicative of ataxin-3 aggregation, in CNT cells that were co-cultured with MJD differentiated neural cells (Fig. 6D, E) compared with CNT cells not co-cultured with MJD cells (Fig. 6B, C). Few mutant ataxin-3 protein nuclear inclusions, spherical intranuclear structures varying in size from 0.5 to ∼6 μm in diameter [1], were detected. Since ataxin-3 microaggregates are neurotoxic and might represent an early step of MJD pathology [18, 19], we quantified the ataxin-3-positive spots/aggregates, as previously done by us [20]. Data revealed a tendency for a progressive accumulation of ataxin-3-positive spots number in CNT cells co-cultured with MJD cells (Fig. 6F). Nevertheless, further experiments are required to confirm this observation.

## Discussion

EVs have been associated with the progression and aggravation of several neurodegenerative diseases contributing to the seed of the aggregation of pathology-associated proteins in illnesses like Alzheimer’s disease and Parkinson’s disease. Regarding MJD, the information considering EVs involvement in neuropathology spreading is very scarce [10]. The present work is the first study evaluating mutant ataxin-3 spreading from MJD patients-derived cells to CNT cells in vitro. Moreover, we investigated two different types of EVs, vesicles produced by iPSC-derived NESC and vesicles produced by their differentiated neural cell cultures. Stem cells are attractive cell models of neurodegenerative diseases; nevertheless, as they have specific metabolic mechanisms and are present in small amounts in the adult brain [21], it is important to also use mature specialized cells to investigate brain diseases. Therefore, in the present study, EVs from both stem cells and their differentiated neural cells, composed of neurons and glia, were used.

A tendency for lower vesicle concentration was detected in MJD Neural-EVs compared to CNT Neural-EVs. This might be explained by the previously reported endosomal pathway deregulation observed in MJD patients. Sittler and colleagues reported higher levels of endosomal markers (Rab7 and Rab1A) and large vesicles accumulating electron-dense materials in brain tissue of MJD patients [22], demonstrating that MJD cells have impairments in the endosomal pathway, which is associated with the production of some EVs, such as exosomes [10]. Autophagy and EVs biogenesis are mechanistically linked by the endolysosomal pathway [23]. Interestingly, the size of Neural-EVs showed a tendency for more homogeneity in the size distribution compared with the NESC-EVs. These results indicate that the type of cells from which the EVs derive play a role in their physical parameters, which is in line with previous reports [10].

Autophagy and cell secretion via EVs act in synergy to protect the cell from external insults maintaining cell homeostasis [24]. Therefore, the presence of autophagy-related proteins in EVs is not surprising. In fact, autophagy-related proteins were also detected in CNT EVs by other authors [25]. As EVs biogenesis and autophagy share molecular mechanisms with notable crosstalk [26], we hypothesize that the presence of autophagy-related proteins in CNT EVs is a result of these shared mechanisms and that cells might be using EVs trafficking to control the levels of the autophagy-related proteins. Moreover, as autophagy is impaired in MJD [7], we observed, as expected, a tendency reduction in the levels of Beclin-1 and p62 in the MJD EVs as compared with CNT EVs, since these proteins are sequestered in protein inclusions in MJD cells [7], with a consequent decrease in the soluble fraction and consequently in the EVs. Thus, our data indicate that EVs from MJD cells transport a lower amount of autophagy-related proteins.

Progenitor cell-derived EVs are known to promote cell survival [27]. Thus, the presence of Akt-1, p-ERK, p-P38, and Bcl-2 cell survival proteins was assessed in the obtained EVs. Akt-1 is an essential intracellular signaling protein regulating cell survival [27], p-ERK plays a vital role in cell proliferation and differentiation [28], p38 regulates cellular processes such as differentiation, growth, and death [29], and Bcl-2 is a pro-survival protein that inhibits apoptosis [30]. With the employed western blot technique, these proteins were not found in NESC-EVs. Further work with more sensitive methods will be necessary to clarify whether these proteins are absent in EVs derived from progenitor cells.

Due to its high energetic demands, the brain exhibits high oxygen use and reactive oxygen species (ROS) production [31]. ROS are kept in check by antioxidants and when this system fails, it can result in neuronal oxidative stress, which is commonly associated with neurodegenerative diseases. Accordingly, in the polyQ Huntington’s disease (HD) it was reported that oxidative damage is increased in *postmortem* tissue from patients and animal models [32, 33]. The accumulation of polyQ proteins also results in the direct production of free radicals [34]. Regarding MJD, it was reported that mutant ataxin-3 decreases antioxidative capacity and increases sensibility to oxidative stress [35], and patients show increased production of ROS [36]. SOD1 is an antioxidant protein that promotes the reduction of cellular ROS levels, which is linked to cell survival under oxidative stress [37]. Our results indicate that this protein is overexpressed in the MJD NESC-EVs as compared with CNT EVs, which can be an attempt of the diseased cells to respond to oxidative stress by increasing SOD1 levels and that is reflected in the content of the secreted EVs.

Parkinson’s disease (PD) is associated with autophagy impairments [38] and PD cells respond to the insult caused by mutant protein accumulation by secreting the mutant protein and mRNA via EVs. Nevertheless, this mechanism results in mutant proteins and RNAs spreading to neighbor cells, aggravating the neuropathology [39, 40]. Moreover, it has been demonstrated that pathological proteins can be transported by EVs [41, 42]. In the present study, no mutant ataxin-3 was detected in the MJD EVs. Nevertheless, this result might reflect the low sensitivity of western blot and RT-PCR to small amounts of protein and mRNA in case these mutant protein and mRNA are secreted to EVs in small amounts.

Strong evidence of the influence of the EVs’ cargo on the gene expression and function of recipient cells has been reported in the last few years. Regarding the impact on oxidative stress, the observed significant induction of ROS in differentiated neural cultures upon incubation over a short period with CNT NESC-EVs was an unexpected result, given that EVs obtained from stem cells, such as NESC, are expected to reduce intracellular ROS [43]. EVs and ROS are interrelated, not only because EVs can produce or detoxify ROS, but also because ROS are involved in the production of EVs [44]. This result might be a consequence of this interplay, namely, of the NADPH oxidase levels and activity; since NADPH oxidase, an enzyme that synthesizes ROS, has been detected in EVs [45]. Nevertheless, further experiments are required to elucidate the mechanism responsible for the increased ROS levels upon EVs administration.

Human differentiated neural cells were treated with 50 and 100 μg/ml of CNT and MJD EVs. The dose of EVs represents 5 and 10 μg of EVs per 100,000 cells, which is in accordance with the 10–25 μg of EVs per 100,000 cells used by other authors [43]. These cells were collected after 3 days (Figs. 4 and 5) or 2 weeks (Fig. S2) of incubation with the EVs. The rationale of these time points was to give time for the EVs and their cargo to influence the protein levels and taking into consideration the half-lives (2–183 h) of the evaluated proteins p62 [46], Beclin-1 [47], LC3B [48], ATG3 [49], ATG7 [50], SOD1 [51].

Data revealed that EVs interfered with the cellular levels of proteins related to autophagy and oxidative stress. MJD NESC-EVs reduced LC3B, ATG3, and ATG7 protein levels after 3 days of incubation, indicating EVs-mediated modifications in autophagy. However, two weeks after the EVs incubation most of these modifications in autophagy impairments were solved by the cells. This might indicate the need for a more permanent exposition of the negative stimulus mediated by EVs to maintain the previously observed autophagy impairments. These autophagy impairments were also observed after the Neural-EVs incubation, triggering a reduction of Beclin-1 and ATG3 protein levels. Intriguingly, p62 protein levels were decreased with MJD EVs and enhanced with CNT EVs. p62 is degraded by autophagy and, therefore, its levels inversely correlate with autophagy activation. However, p62 changes can also occur independently of autophagy [52], because this protein is involved in several other cellular processes, such as apoptosis and inflammation [53]. Thus, we hypothesize that other factors delivered by EVs are interfering with other mechanisms regulated by p62.

It was previously reported that early/moderate-stage MJD patients present a decreased antioxidant capacity and increased ROS generation [36] and that the administration of EVs secreted from cells under oxidative stress affects the state of oxidative stress in recipient cells [54]. Accordingly, the present study evaluated the impact of MJD EVs on SOD1 levels of the recipient cells. SOD1 protein levels were found decreased by MJD NESC-EVs after 2 weeks and by MJD Neural-EVs after 3 days of incubation. CNT EVs promote no SOD1 decrease; on the contrary, a tendency for enhancement was observed. This data clearly demonstrated that MJD EVs can interfere with and deregulate cell defense mechanisms, namely autophagy and antioxidant protein levels.

Regarding the mutant ataxin-3 spreading evaluation, MJD and CNT differentiated neural cultures were grown during 1, 3, and 8 weeks in conditions without cellular contact and evaluated for the presence of mutant ataxin-3 inclusions at these time points. The CNT cells that shared culture media with MJD cells exhibited some mutant ataxin-3 aggregates and a tendency for increased mutant ataxin-3-positive spots in the cell nucleus. The presence of protein inclusions is reported to be implicated in the progression of age-associated diseases, such as MJD [10, 55]. Moreover, it has also been reported that disease-spreading mechanisms are dependent on the time of exposure [10, 56]. Therefore, we hypothesize that future studies with CNT and MJD cells co-cultured for more than 8 weeks might increase the number of mutant ataxin-3 protein inclusions observed in CNT cells. Finally, the sharing of cell culture medium between CNT and MJD cells might result in disease-spreading between cells through other mechanisms besides extracellular vesicles, namely by soluble oligomers of the pathological proteins that can evade the cell and disseminate to adjacent cells [10]. Our data indicate potential mutant ataxin-3 spreading from MJD to CNT cells, nevertheless, a more complete study is required to further investigate this mechanism.

In conclusion, in this work, EVs obtained from human CNT and MJD iPSC-derived NESC (CNT and MJD-EVs) and from their differentiated neural cultures (CNT and MJD Neural-EVs) were isolated and characterized. The autophagy-related proteins p62 and Beclin-1 and the free radical scavenging protein SOD1 were detected in NESC-EVs and Neural-EVs. Regarding the mRNA cargo, the autophagy-related *SQSTM1*, *BECN1*, *UBC*, *ATG12*, and *LC3B* mRNAs, and the oxidative stress-related *CYCS* mRNA were detected both in CNT and MJD NESC-EVs. Incubation with MJD NESC-EVs or MJD Neural-EVs resulted in the reduction of autophagy-related proteins and SOD1 protein levels in CNT cells. Finally, in an indirect co-culture model, CNT cells that shared culture media with MJD cells showed a tendency for increased mutant ataxin-3-positive protein spots in the cell nucleus, indicating potential mutant ataxin-3 spreading from MJD to CNT cells.

Altogether, this work provides evidence of EVs-mediated interference in MJD-associated neuropathology mechanisms, namely in autophagy and oxidative stress.

## Materials and methods

### Human CNT and MJD iPSC-derived neuroepithelial stem cells (NESC)

Human CNT and MJD iPSC-derived Neuroepithelial Stem Cells (NESC) were established and extensively characterized in our lab [57]. Briefly, fibroblasts of CNT and MJD patients were reprogrammed into iPSC using lentivirus encoding for four reprogramming factors (Oct-4, Klf4, c-Myc, and Sox-2) [58, 59]; then iPSC were induced into NESC [60]. NESC were cultured as monolayers in Matrigel (hESC-qualified Matrix LDEV-Free, BD Matrigel, Corning)-coated flasks in maintenance culture medium composed by N2B27 medium (Neurobasal medium (Invitrogen) and DMEM/F-12 (Invitrogen), in a 1:1 ratio, 1% Penicillin/Streptomycin (Invitrogen), 2 mM L-Glutamine (Invitrogen), 1:200 N2 supplement (Gibco), and 1:100 B27 without vitamin A (Gibco)), supplemented with 150 μM Ascorbic Acid (Sigma-Aldrich), 3 μM CHIR 99021 (Axon Medchem), and 0.75 μM Purmorphamine (PMA) (Enzo) at 37 °C with 5% CO_2_. Cells were split every 5–7 days. Cells are periodically tested for mycoplasma contamination. The culture medium to isolate NESC-EVs was collected immediately before splitting the cells and kept at −20 °C until EVs isolation.

### Neural cells differentiated from iPSC-derived NESC (differentiated neural cells)

The human CNT and MJD iPSC-derived NESC were differentiated into neural cells constituted by neurons and glia in Matrigel-coated T75 flasks for the production of culture medium for isolation of Neural-EVs, and in Matrigel-coated cell culture plates (MW6, MW12, and MW96) for cell assays. Cells were plated in the differentiation culture medium composed of N2B27 medium supplemented with 0.25 μM Dibutyryl cyclic Adenosine Monophosphate sodium salt (cAMP) (Sigma-Aldrich), 5 μM Forskolin (Sigma-Aldrich), and 2 μM Retinoic Acid (Sigma-Aldrich) [61]. Cells were differentiated for 7 days before being used in the assays. Differentiation media was changed every 3–4 days in plates and flasks. The culture medium used to isolate Neural-EVs was collected every 3–4 days and stored at −20 °C until EVs isolation.

### EVs isolation by differential centrifugation

The culture medium of iPSC-derived NESC and differentiated neural cells was collected and stored at −20 °C until EVs isolation. The culture medium was defrosted overnight at 4 °C, and then the medium was centrifuged at 300 × *g* for 10 min at 4 °C to pellet cells. Subsequently, the supernatant was centrifuged at 2000 × *g* for 10 min at 4 °C to remove the remaining cells and cell debris. The resulting supernatant was filtered using a 0.8 μm cellulose acetate filter (Whatman) to remove larger vesicles. Finally, the medium was ultracentrifuged at 10,000 × *g* for 1 h and 30 min at 4 °C, using the SW-41Ti rotator, in the Optima XE-100 ultracentrifuge (Beckman Coulter). The resulting pellet was resuspended and stored according to the final use of the sample: for western blot analysis, the pellet was resuspended in lysis buffer and kept at −80 °C; for RNA analysis, lysis buffer (provided by miRCURY™ RNA Isolation kit, Qiagen) and 1% β-mercaptoethanol was used and kept at −80 °C; and for Nanoparticle Tracking Analysis (NTA) and functional evaluation it was resuspended in sterile PBS and kept at −80 °C and 4 °C, respectively. EVs were quantified for the total protein using Pierce BCA Protein Assay Kit (Thermo Fischer Scientific).

### Nanoparticle tracking analysis (NTA)

NTA was performed using a NanoSight NS300 instrument equipped with a 488 nm laser and an sCMOS camera module (Malvern Panalytical Limited, Malvern, UK) following general recommendations. EVs isolated as previously described were resuspended in 100 μL PBS after ultracentrifugation and, immediately before analysis, diluted in particle-free PBS before loading. If necessary, samples were further diluted to obtain the optimum concentration range. Readings were performed under constant flow, and settings were optimized and kept constant to determine the mean, median, and mode size, and the estimated concentration of particles (injection speed set to 20, camera level was 12–14). Five technical replicates were performed for each sample (60 s each). Data were processed using NTA 3.3 (Dev Build 3.3.301) analytical software (Malvern Panalytical Limited, Malvern, UK), with a Detection Threshold of 3.

### Transmission electron microscopy (TEM)

Transmission electron microscopy was performed according to Théry et al. [62]. Briefly, after isolation, EVs were fixed with 2% paraformaldehyde (PFA) and deposited on Formvar-carbon coated grids (TAAB Laboratories Equipment) for 20 min. Grids were washed with PBS and fixed for 5 min with 1% glutaraldehyde. Following a cycle of washes using distilled water, grids were contrasted with a uranyl-oxalate solution (pH = 7) for 5 min and transferred to methyl-cellulose-uranyl acetate for 10 min on ice. Images were obtained using a Tecnai G2 Spirit BioTWIN electron microscope (FEI) at 80 kV.

### EVs labeling with fluorescence CFSE probe

After isolation, as previously described, the EVs pellet was resuspended in PBS. Then 10 μM of Carboxyfluorescein Diacetate Succinimidyl Ester (CFSE) (CellTrace, Invitrogen) in DMSO was added to fluorescently label the vesicles. CFSE is a non-fluorescent molecule that easily diffuses across cell membranes and once inside cells/cellular vesicles, the acetate groups are cleaved by esterases yielding a fluorescent molecule that interacts with primary amines of intracellular/intravesicular proteins to form stable amide bonds [63, 64]. CFSE incubation was done for 45 min at 37°C, with shaking every 15 min. Subsequently, EVs were diluted in 9.5 mL of PBS and ultracentrifuged at 100,000 × *g* for 1 h and 30 min, at 4 °C, to remove the unbound dye. In parallel, a negative CNT (CFSE + PBS, without EVs), composed of the same amount of CFSE was added to 9.5 mL of PBS and submitted to the same steps as the CFSE-labeled EVs sample. After ultracentrifugation, both samples were resuspended in 100 μL of sterile PBS. The obtained CFSE-labeled EVs were quantified for the total protein using Pierce BCA Protein Assay Kit and stored at 4 °C until administration to the cells.

### Cell internalization of CFSE-labeled EVs

CNT iPSC-derived NESC plated in Matrigel-covered coverslips at the density of 250,000 cells per well (in 12-well plates) in 500 µl of differentiation culture medium were differentiated for 1 week, as previously described. Cells were incubated for 14 h with 50 μg/ml of CNT NESC-EVs and CNT Neural-EVs labeled with CFSE, as previously described. Then, cells were collected, washed twice with PBS, fixed with 4% paraformaldehyde (PFA) for 20 min at room temperature, and stored at 4 °C until being processed for immunocytochemistry.

### Indirect co-culture of CNT and MJD differentiated neural cells

CNT iPSC-derived NESC were plated in Matrigel-covered coverslips in 12-well plates, 300,000 cells per well. MJD iPSC-derived NESC were plated in cell culture inserts (1.0 µm, Polyethylene Terephthalate, 12-well) (Millicell) also covered with Matrigel, 200,000 cells per insert. CNT and MJD cells were grown, sharing the same differentiation culture medium through the 1.0 μm pore of the insert membrane, for 1, 3, or 8 weeks. Cells were collected after this time points. For experiment CNT, the CNT iPSC-derived NESC were cultured without sharing culture medium with other cells.

### Reactive oxygen species (ROS) measurement

Human iPSC-derived NESC were plated in differentiation culture medium and Matrigel-covered plates, as previously described. It was plated 75,000 cells per well in 200 µl of culture medium in flat bottom clear, black polystyrene 96-well plates (Corning). After 7 days of differentiation, cell cultures were washed twice with Hanks’ Balanced Salt Solution (HBSS) (Sigma-Aldrich) and incubated with 100 μg of CNT and MJD NESC-derived EVs, in 200 μl of HBSS, for 1 h at 37 °C and 5% CO_2_ for ROS induction. Afterward, the Reactive Oxygen Species Detection Assay Kit (Abcam) was used according to the manufacturer´s instructions. Briefly, the probe was incubated with the cells for 2 h and the red fluorescence was determined in the Cytation Cell Imaging Reader (Agilent BioTek) with an excitation wavelength of 520 nm, emission of 605 nm, and cutoff of 590 nm. The medium intensity fluorescence for each condition was determined.

### Cell viability assay

Human iPSC-derived NESC were plated in differentiation culture medium, 1 × 10^6^ cells per well (in 6-well plates) in 1 mL, growing in Matrigel-covered plates, as previously described. Cell viability was assessed 3 days after incubation with 50 and 100 µg/ml EVs with the resazurin reduction assay, which measures the chemical reduction of the resazurin dye resulting from cell metabolic activity. Briefly, cells were incubated with 0.1 mg/mL resazurin, prepared in the differentiation culture medium, for 4 h at 37 °C and 5% CO_2_. The absorbance of the resazurin reduced and oxidized species was determined at 570 nm and 600 nm, respectively, using a spectrophotometer (SpectraMax Plus 384, Molecular Devices). Cell viability was calculated according to the equation: [(A_570_ – A_600_) test group cells × 100)/(A_570_ – A_600_) CNT cells].

### Ataxin-3 aggregation quantification

Cell cultures were labeled for ataxin-3 through immunocytochemistry, as previously described [20]. Five pictures of each condition in each independent experiment were acquired with Plan-Apochromat 63x/1.40 Oil DIC M27 objective and ApoTome.2 on a Carl Zeiss Axio imager Z2 microscope. Using the Icy bioimage informatics platform [65], pictures were quantified for ataxin-3-positive spots/aggregates present in the cell nucleus (ROI created from 4’,6-diamidino-2-phenylindole (DAPI) channel). The settings regarding ataxin-3-positive spots object size and sensitivity were kept constant in all quantified pictures. Finally, the number of ataxin-3-positive aggregates and DAPI area (mm^2^) were automatically determined for each picture using the Icy bioimage informatics platform, and the number of ataxin-3-positive aggregates was normalized to the DAPI area in each image [20].

Quantitative RT-PCR, Semi-Quantitative RT-PCR, Immunocytochemistry, and Western Blot (full-length uncropped original western blots are provided in Supplemental Material) were performed as previously described [61, 66]. Detailed protocol in Supplementary Materials and Methods.

### Microscopy acquisition

Images were acquired at room temperature with an Axio Imager Z2 widefield microscope (CCD monochromatic digital camera Axiocam HRm) using EC Plan-Apochromat 10x/0.3NA or Plan-Apochromat 20x/0.8NA air objectives and Plan-Apochromat 63x/1.40 Oil DIC M27 objective and ApoTome.2. Confocal fluorescence images were obtained with an LSM 710, Axio Observer using Plan-Apochromat 40x/0.8NA and Plan-Apochromat 63x/1.4NA objective.

### Statistical analysis

Data are presented as mean ± SEM. Outliers were identified with Grubbs’ test (significance level 0.05) and excluded from the analysis. Differences in the mean between two groups were analyzed using the Unpaired t*-*test with Welch’s post hoc test; to compare means between more than two groups, a One-way analysis of variance (ANOVA) with Tukey’s post hoc test was used. Graphs and statistical analysis, as indicated in each figure legend, were performed in GraphPad Prism software, *P* < 0.05 was considered to be statistically significant.

## Supplementary information

Supplementary Information

Uncropped western blot membranes

## Data Availability

The data generated during and/or analyzed during the current study are available from the corresponding author upon reasonable request.
