# Supplementary material for: Autophagy- and oxidative stress-related protein deregulation mediated by extracellular vesicles of human MJD/SCA3 iPSC-derived neuroepithelial stem cells and differentiated neural cultures
Source: Cell Death Dis. 2025 May 15;16(1):383. doi: 10.1038/s41419-025-07659-0 (PMC12081669; doi:10.1038/s41419-025-07659-0)
Supplement: Supplementary file 1 — Supplementary Information [file 41419_2025_7659_MOESM1_ESM.docx]

**Autophagy- and oxidative stress-related protein deregulation mediated by extracellular vesicles of human MJD/SCA3 iPSC-derived neuroepithelial stem cells and differentiated neural cultures**

Liliana S. Mendonça^1,2,3,&,^*, Ricardo Moreira^1,2,4,&^, Daniel Henriques^1,2,3^, Mónica Zuzarte^4,5,6^, Teresa M. Ribeiro-Rodrigues^2,5,6^, Henrique Girão^2,5,6^, Luís Pereira de Almeida^1,2,3,4,^*

**Supplementary Information**

**Supplementary Material and Methods**

**Quantitative Reverse Transcriptase (RT) - Polymerase Chain Reaction (PCR) and Semi-Quantitative RT-PCR**

RNA was extracted from EVs isolated from 50 mL of conditioned media of iPSC-derived NESC or differentiated neural cultures, as previously described, using miRCURY™ RNA Isolation kit (Quiagen), according to the manufacturer’s recommendations. The RNA was eluted with 30 μl of DNase and RNase free water (Sigma) and purity and concentration were measured using NanoDrop™ 2000 (Thermo Scientific). The RNA was kept at -80ºC until further processing. Then, cDNA was synthesized from 300 ng of total RNA with iScript™ cDNA Synthesis Kit (Bio-Rad), as instructed by the manufacturer. Quantitative real-time PCR (qRT-PCR) was performed with SsoAdvanced™ SYBR® Green Supermix Kit (Bio-Rad). Briefly, qRT-PCR was performed with a single cycle of 95ºC for 30 seconds, followed by 45 cycles of two steps: a first step of 5 seconds at 95ºC, and a second step of 15 seconds at a temperature depending on the annealing temperature of each primer (Table S1). The threshold cycle (CT) for each gene was generated automatically by the StepOne™ Software (Applied Biosystems). For all genes, a standard curve was performed, and quantitative PCR efficiency was determined by the software. Additionally, no template and no reverse transcriptase negative CNTs were included. The relative mRNA quantification concerning CNT samples was determined by the Pfaffl method, taking into consideration the different amplification efficiencies of all genes, and GAPDH was used as housekeeping gene.

Semi-quantitative RT-PCR was performed using 40 ng of cDNA obtained as described above. The reaction mix used was as follows: 1x GC buffer, 0.2 mM dNTP, 500 nM of each forward and reverse primers, 5% DMSO, 1% Phusion® enzyme (Quiagen) in DNase-free water (Sigma). The PCR was performed with a single cycle of 98ºC for 10 seconds followed by 35 cycles of 3 steps: 1) 98ºC for 10 seconds; 2) 10 seconds at variable temperature depending on the primer’s annealing temperature (Table S1); 3) 72ºC for 30 seconds. This was followed by a final single cycle of 72ºC for 30 minutes. Samples were kept at 4ºC until agarose gel electrophoresis resolution. Then, samples were loaded in 2% agarose gel and electrophoresis was performed at 90 V for 45 minutes to 1 hour. Images were taken using Image Lab software (Bio-Rad).

**Western blot**

Human iPSC-derived NESC plated in Matrigel-covered plates at 1x10^6^ cells per well (in 6-well plates) in 1mL of differentiation culture medium, as previously described, and after 7 days of differentiation were incubated with 50 and 100 µg/ml EVs. Three days or 2 weeks after EVs incubation, cells were collected, washed with PBS, and stored at -80ºC until further processing. EVs isolated from 50 mL of conditioned media of iPSC-derived NESC or differentiated neural cultures, as previously described, were kept at -80ºC until processing.

Cell and EVs were disrupted with 100 μL lysis buffer composed of 150 mM NaCl, 50 mM Tris, 5 mM EDTA, 1% Triton, 0.5% sodium deoxycholate, and 0.1% SDS freshly added with protease inhibitor (Complete Mini), phosphatase inhibitor (PhosStop Easy, Roche), 1 mM Phenylmethane Sulfonyl Fluoride (PMSF) (Sigma-Aldrich), and 10 μg/mL Dithiothreitol (DTT) (Sigma-Aldrich), strong vortex, and 3 sonication cycles of 10 seconds at 40-60 kHz.

For each sample, protein quantification was done with Pierce BCA Protein Assay Kit (Thermo Fischer Scientific). Samples with 50 μg of protein were prepared with sample buffer (0.5 M Tris-HCl, pH 6.8, 30% glycerol, 10% SDS, 0.6 M DTT, and 0.1 mg/mL blue bromophenol) denatured by heating at 95ºC for 5 minutes and stored at -20ºC until use. Samples were loaded and resolved in SDS-polyacrylamide gel electrophoresis (PAGE) (4% of loading gel and 10% or 12% of acrylamide of resolving gel for proteins over 50 KDa and under 50 KDa, respectively), resolving was done at 70V for 10-15 minutes and 100V for the rest of the run in Bicine buffer. After electrophoresis, proteins were transferred to polyvinylidene difluoride (PVDF) membranes (Immobilon-P Membrane, Millipore) at 0.75 A for 2 to 2:30 hours at 4ºC in (N-cyclohexyl-3-aminopropanesulfonic acid) CAPS/methanol buffer. Then, the membranes were blocked with 5% milk in TBS (Tris-Buffered Saline) with 0.1% Tween 20 (Sigma) (TBS-T) for 1 hour and incubated overnight at 4ºC with primary antibodies (Table S2) in 5% milk in TBS-T. Afterward, membranes were washed three times (5, 10, and 15 minutes) with TBS-T and incubated for 2 hours at room temperature with alkaline phosphatase-linked secondary antibodies (Table S2) in 5% milk in TBS-T. Then, the membranes were washed with TBS-T three times (5, 10, and 15 minutes) and incubated with Enhanced Chemifluorescence substrate (ECF, Amersham Biosciences) for up to 5 minutes. The signal was detected in the Versadoc 3000 Imaging System (Bio-Rad) and quantified using ImageJ software (NIH, USA) and normalized for β-tubulin and the CNT, as indicated in the figure’s legends.

**Immunocytochemistry**

Human iPSC-derived NESC plated in Matrigel-covered coverslips (12-well plates), were washed twice with PBS, fixed with 4% PFA (Sigma) for 20 minutes at room temperature, washed again twice with PBS, and stored at 4ºC until use. Then, cells were permeabilized with 1% Triton™ X-100 (Sigma) for 5 min at room temperature, followed by 1 hour blocking with 3% bovine serum albumin (BSA) (Sigma) in PBS at room temperature. Afterward, cells were incubated with primary antibodies (Table S2) prepared in 3% BSA/PBS, overnight at 4ºC. Subsequently, coverslips were washed twice with PBS and incubated with the secondary antibodies anti-rabbit Alexa Fluor-488 and anti-mouse Alexa Fluor-594 (1:200, ThermoFisher), in 1% BSA/PBS, for 2 hours at room temperature. Nuclear DNA was stained blue with 4’, 6-diamidino-2-phenylindoline (DAPI) (1:5000, AppliChem). Coverslips were washed three times with PBS and mounted in glass microscope slides with Mowiol reagent (Sigma). After drying for 15 minutes at 37ºC were stored at 4ºC until microscopy observation.

**Supplementary Tables**

**Table S1. Primers for RT-PCR and qRT-PCR**

| \| Gene \| Brand \| Reference/sequence \| Annealing temperature \| \| --- \| --- \| --- \| --- \| \| SQSTM1 \| Sigma \| KPP; SY160445847084F: TGTGAATTTCCTGAAGAACG SY160445847085R: TCGATATCAACTTCAATGCC \| 56ºC \| \| BECN1 \| Sigma \| KPP; SY171042912049F: CAGTATCAGAGAGAATACAGTG  SY171042912050R: TGGAAGGTTGCATTAAAGAC \| 60ºC \| \| UBC \| Sigma \| KPP; SY171211736068F: CGTCACTTGACAATGCAG SY171211736069R: TGTTTTCCAGCAAAGATCAG \| 60ºC \| \| ATG12 \| Sigma \| KPP; SY171211736070F: CTCTCTATGAGTGTTTTGGC SY171211736071R: CACATCTGTTAAGTCTCTTGC \| 56ºC \| \| CYCS \| Sigma \| KPP; SY170906722054F: AAGAACAAAGGCATCATCTG SY170906722055R: GCTATTAAGTCTGCCCTTTC \| 58ºC \| \| GAPDH \| Sigma \| KPP; SY200128161070F: ACAGTTGCCATGTAGACC SY200128161071R: TTGAGCACAGGGTACTTTA \| 56ºC \| \| LC3B \| Sigma \| KPP; SY160445847090F: ATAGAACGATACAAGGGTGAG SY160445847091R: CTGTAAGCGCCTTCTAATTATC \| 59ºC \| \| ATXN3 \| Invitrogen \| Forward: GGAAGAGACGAGAAGCCTACTT  Reverse: GCATCA CCTAGATCACTCCCA \| 58ºC \| |
| --- | --- | --- | --- | --- | --- | --- | --- | --- | --- | --- | --- | --- | --- | --- | --- | --- | --- | --- | --- | --- | --- | --- | --- | --- | --- | --- | --- | --- | --- | --- | --- | --- | --- | --- | --- | --- |
| KPP: KiCqStart Pre-designed Primers |

**Table S2. Antibodies Information**

| \| Antibody \| Dilution \| Brand \| Reference \| Protein Molecular Weight \| Specie \| \| --- \| --- \| --- \| --- \| --- \| --- \| \| Alix (3A9) \| 1:1000 \| Cell Signaling \| 2171S \| 96 kDa \| mouse \| \| Akt1 \| 1:500 \| Santa Cruz \| sc-5298 \| 62 kDa \| mouse \| \| Ataxin-3 \| 1:1000 \| Millipore \| MAB5360 \| 50 (wild type), 66 (mutant) kDa \| mouse \| \| ATG3 \| 1:500 \| Cell Signaling \| 3415S \| 37 kDa \| rabbit \| \| ATG7 \| 1:500 \| Cell Signaling \| 8558S \| 77 kDa \| rabbit \| \| Beclin-1 \| 1:1000 \| BD Biosciences \| 612113 \| 61 kDa \| mouse \| \| Bcl-2 \| 1:500 \| Cell Signaling \| 2876S \| 26 kDa \| rabbit \| \| β-tubulin \| 1:1000 \| Sigma \| SAP.4G5 \| 50 kDa \| mouse \| \| β3-tubulin \| 1:400 \| Invitrogen \| 32-2600 \| Used in immunolabeling \| Mouse \| \| Calnexin \| 1:500 \| Santa Cruz \| sc-11397 \| 90 kDa \| rabbit \| \| LC3B \| 1:1000 \| Cell Signaling \| 2775S \| 18 kDa \| rabbit \| \| p-ERK \| 1:500 \| Santa Cruz \| sc-7383 \| 43 kDa \| mouse \| \| p-p38 \| 1:500 \| Santa Cruz \| sc-7973 \| 38 kDa \| mouse \| \| p62 \| 1:1000 \| Cell Signaling \| 5114S \| 62 kDa \| rabbit \| \| SOD1 \| 1:500 \| Santa Cruz \| sc-17767 \| 16-18 kDa \| mouse \| \| Anti-mouse IgG, IgM (H+L) Secondary Antibody, AP \| 1:10 000 \| Invitrogen \| 31328 \| Used in western blot \| goat \| \| Anti-rabbit IgG (H+L) Secondary Antibody, AP \| 1:10 000 \| Invitrogen \| 31340 \| Used in western blot \| goat \| \| Anti-mouse Alexa 568 Secondary Antibody \| 1:250 \| Invitrogen \| A10042 \| Used in immunolabeling \| donkey \| \| Anti-rabbit Alexa 488 Secondary Antibody \| 1:250 \| Invitrogen \| A11015 \| Used in immunolabeling \| donkey \| |
| --- | --- | --- | --- | --- | --- | --- | --- | --- | --- | --- | --- | --- | --- | --- | --- | --- | --- | --- | --- | --- | --- | --- | --- | --- | --- | --- | --- | --- | --- | --- | --- | --- | --- | --- | --- | --- | --- | --- | --- | --- | --- | --- | --- | --- | --- | --- | --- | --- | --- | --- | --- | --- | --- | --- | --- | --- | --- | --- | --- | --- | --- | --- | --- | --- | --- | --- | --- | --- | --- | --- | --- | --- | --- | --- | --- | --- | --- | --- | --- | --- | --- | --- | --- | --- | --- | --- | --- | --- | --- | --- | --- | --- | --- | --- | --- | --- | --- | --- | --- | --- | --- | --- | --- | --- | --- | --- | --- | --- | --- | --- | --- | --- | --- | --- | --- | --- | --- | --- | --- | --- |

**Supplementary Figures**

**
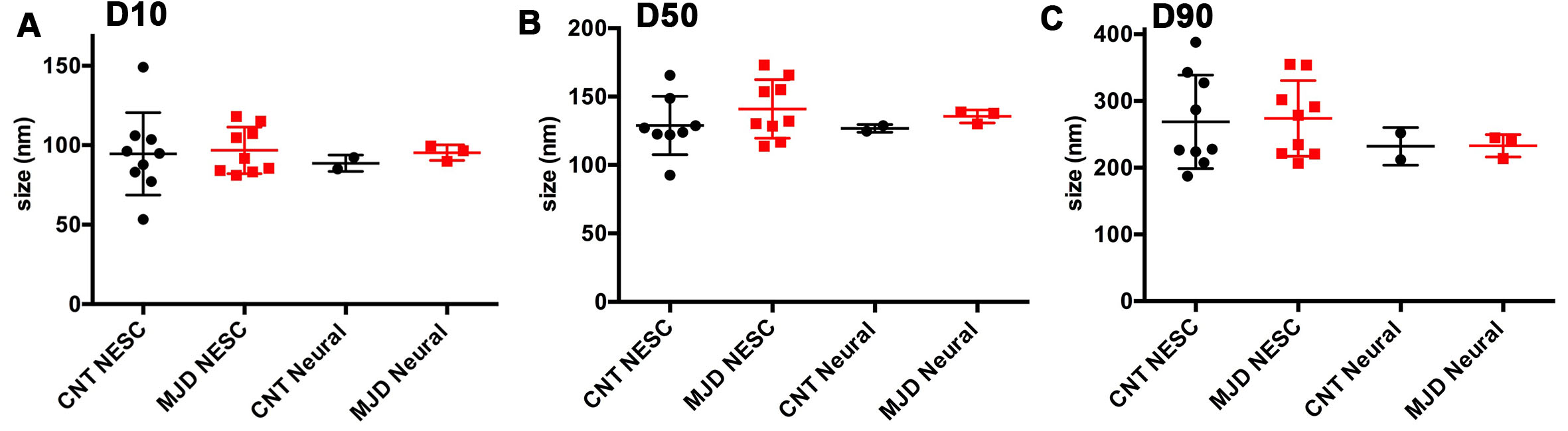
**

**Fig. S1. Percentile distribution (D10, D50, D90) of MJD and CNT EVs’ size.** The size of EVs isolated from the culture media of CNT and MJD iPSC-derived NESC (CNT NESC and MJD NESC) and neural cell cultures differentiated from iPSC-derived NESC (CNT Neural and MJD Neural) was measured with Nanoparticle Tracking Analysis (NTA), as described in Figure 1, and the percentile distribution of (**A**) 10, (**B**) 50, and (**C**) 90 % of the vesicles was evaluated. Unpaired t-test with Welch’s correction, NESC-EVs n=7-8, Neural-EVs n=3.

**
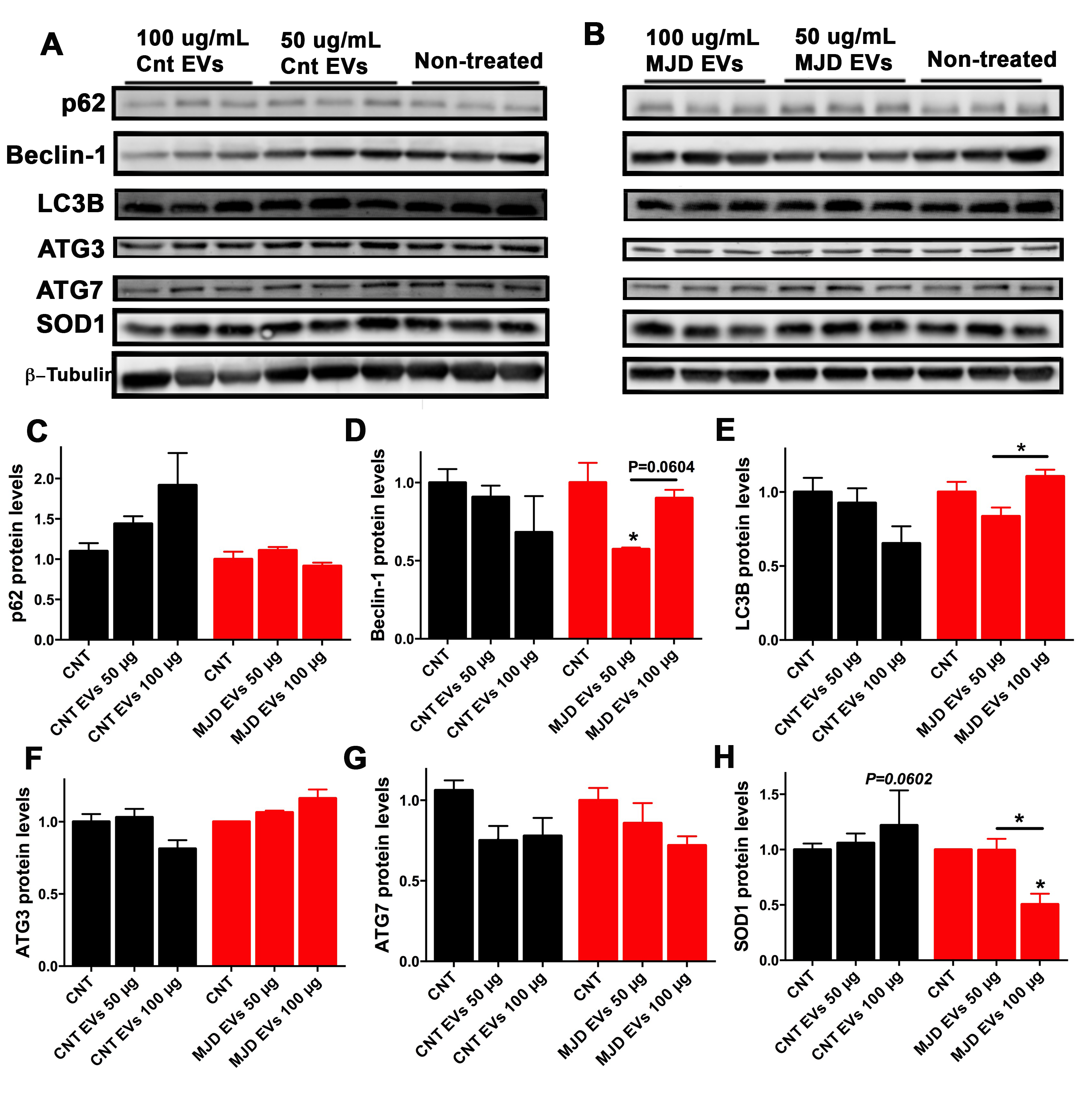
Fig. S2. Effect of CNT and MJD NESC-EVs in the levels of proteins related to autophagy and oxidative stress after 2 weeks.** (**A-H**) Western blot analysis of proteins related to autophagy (p62, Beclin-1, LC3B, ATG3, and ATG7) and oxidative stress (SOD1) in differentiated neural cell cultures after 2 weeks incubation with 50 and 100 μg/ml of CNT (CNT EVs) and MJD (MJD EVs) NESC-EVs and non-treated cells (CNT, non-treated). Representative Western blot images of p62, Beclin-1, LC3B, ATG3, ATG7, SOD1, and β-Tubulin protein levels after incubation with (**A**) CNT and MJD (**B**) NESC-EVs. Quantification of (**C**) p62, (**D**) Beclin-1, (**E**) LC3B, (**F**) ATG3, (**G**) ATG7, and (**H**) SOD1 protein levels in human differentiated neural cell cultures incubated with 50 and 100 μg/ml (50 and 100 μg) of CNT (CNT EVs) and MJD (MJD EVs) NESC-EVs, normalized for non-treated cells (CNT); n=3. Data are expressed as mean ± SEM, *p<0.05, One-way ANOVA with Tukey’s multiple comparison test.
