# Supplementary figures and images for: Autophagy- and oxidative stress-related protein deregulation mediated by extracellular vesicles of human MJD/SCA3 iPSC-derived neuroepithelial stem cells and differentiated neural cultures
Source: Cell Death Dis. 2025 May 15;16(1):383. doi: 10.1038/s41419-025-07659-0 (PMC12081669; doi:10.1038/s41419-025-07659-0)

Figure 1:

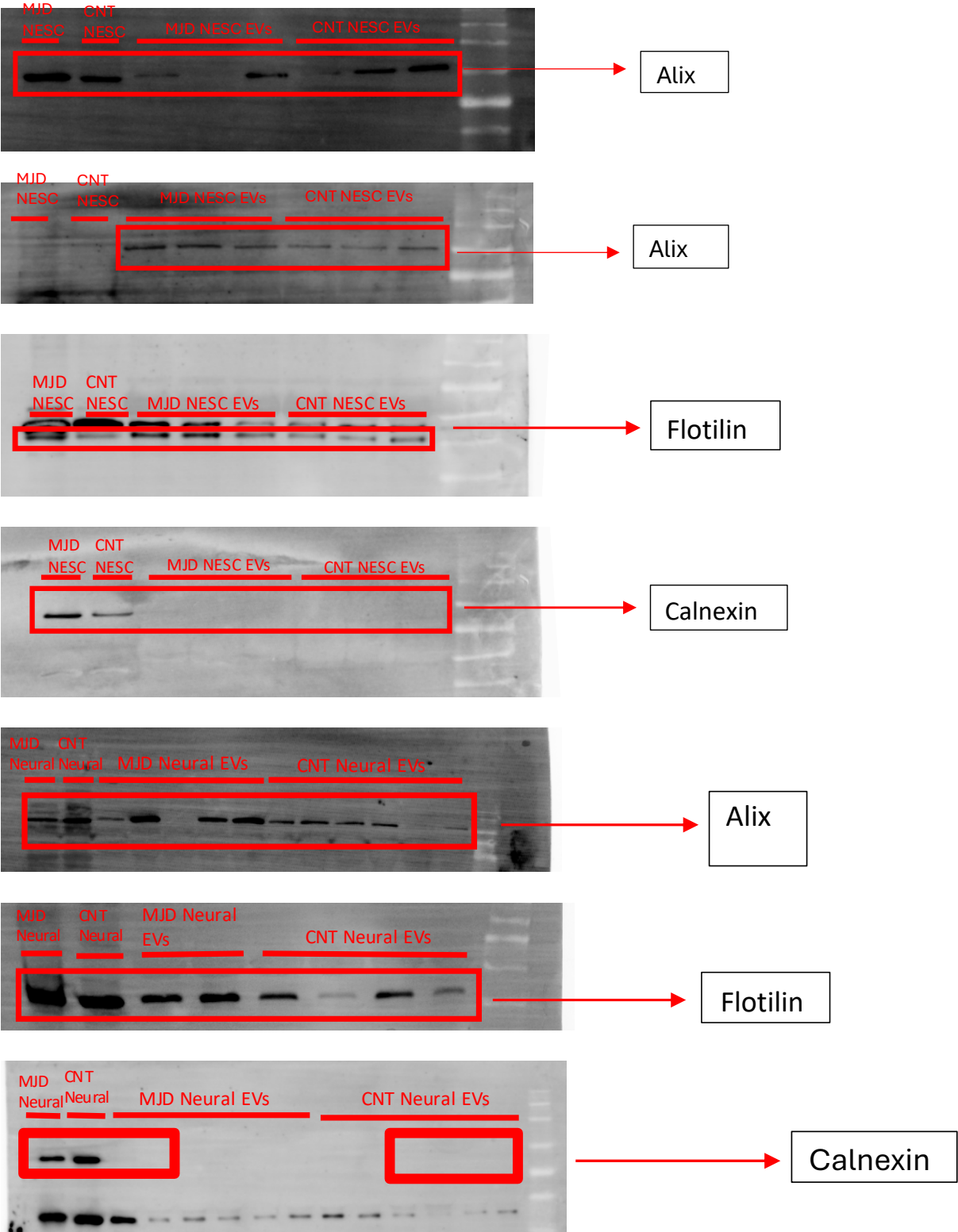

Figure 2:

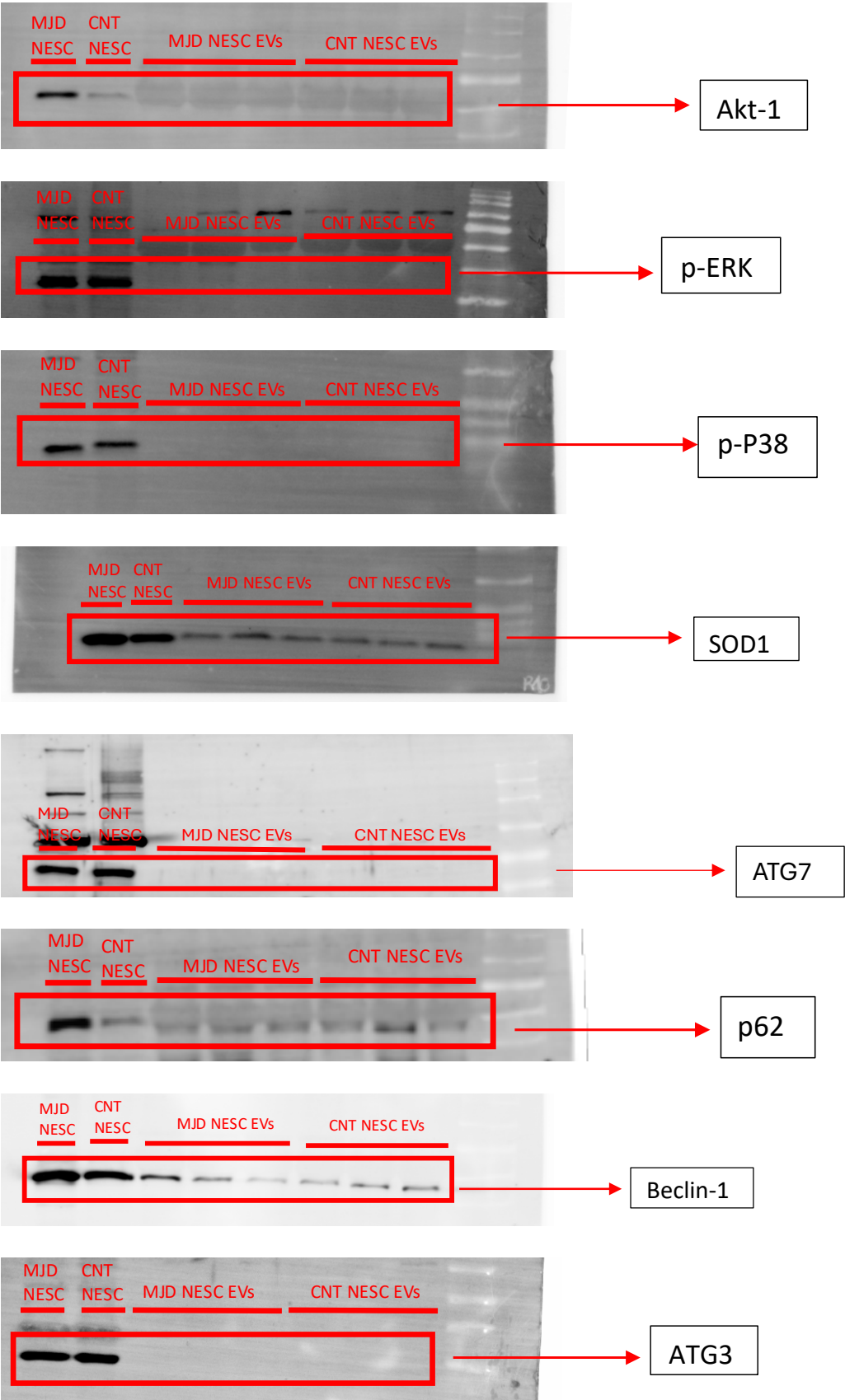

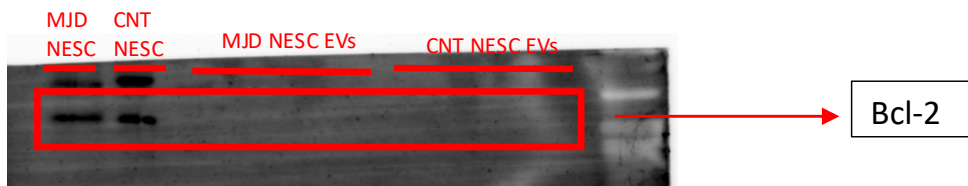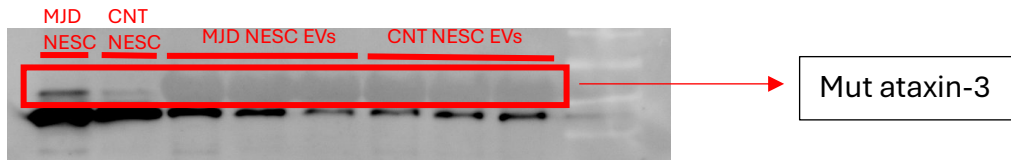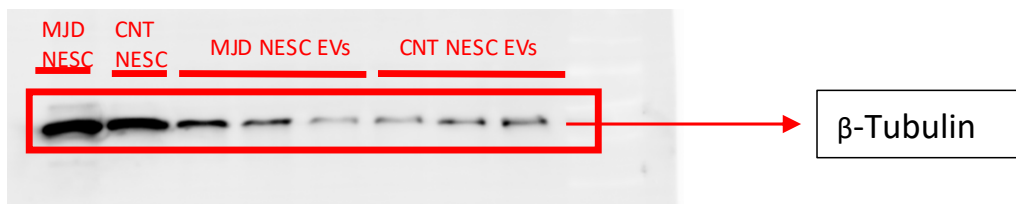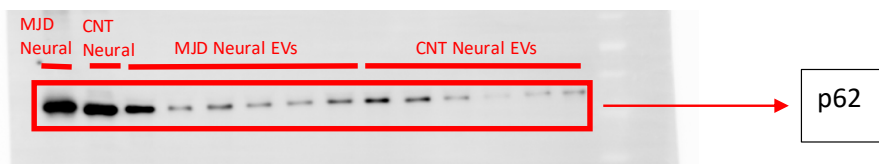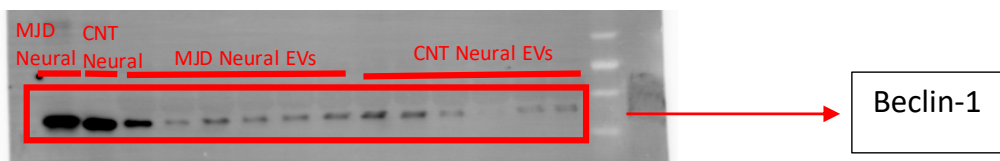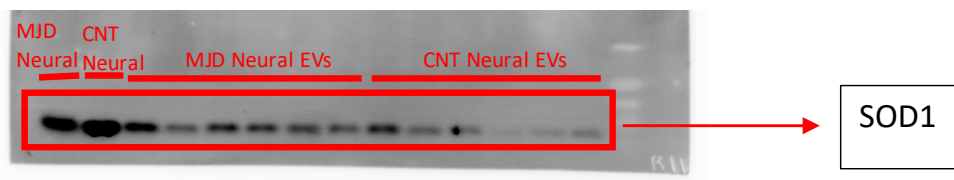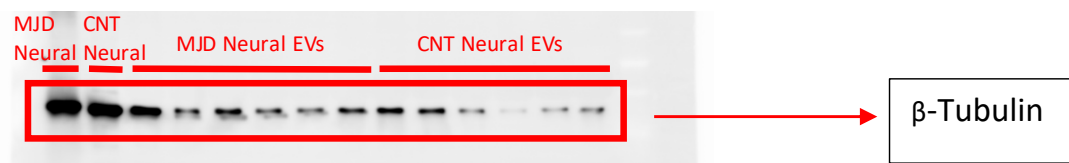

**Figure 4:**

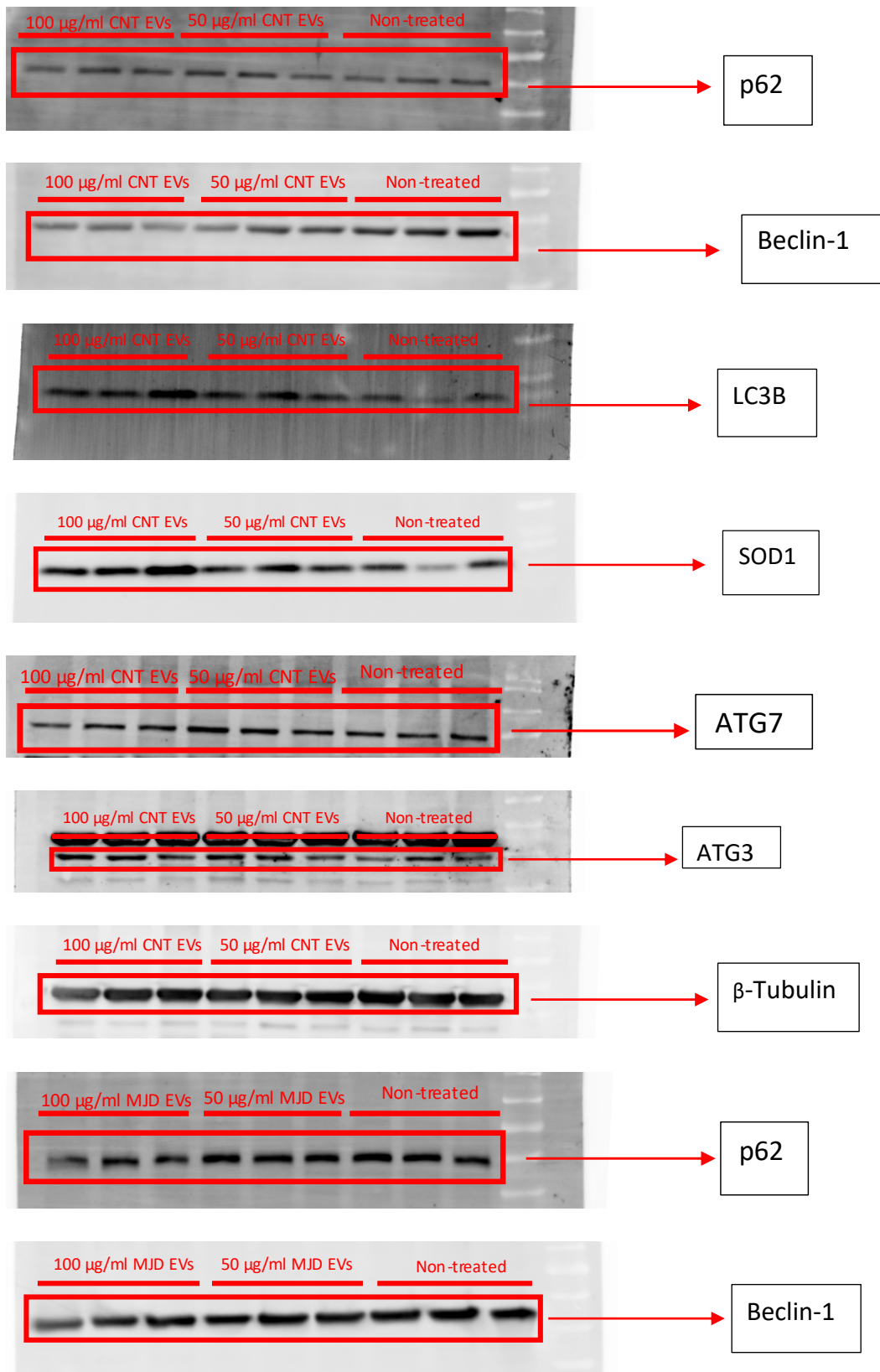

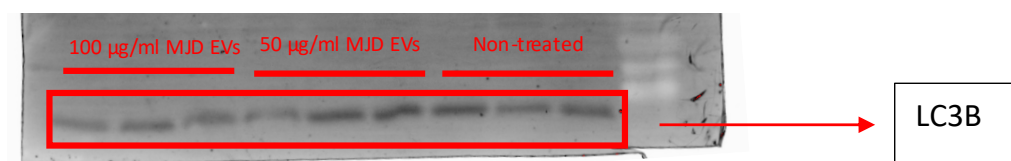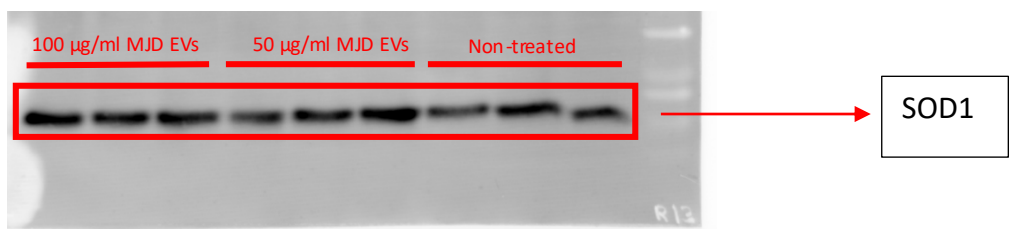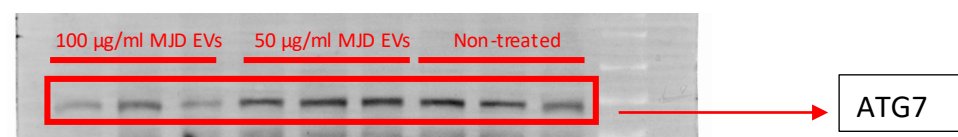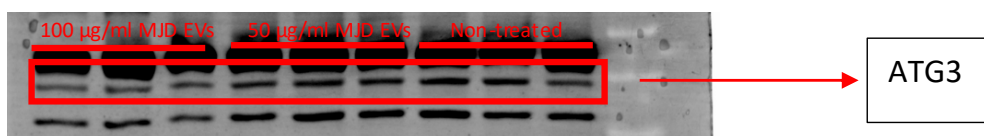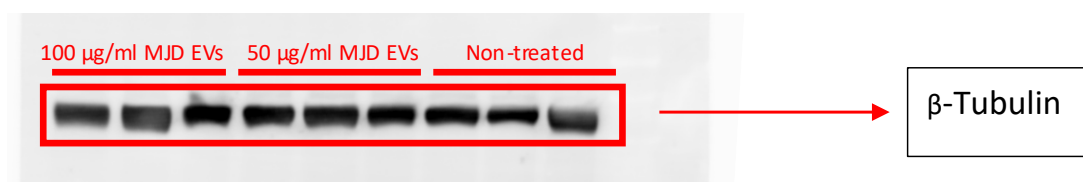

**Figure 5:**

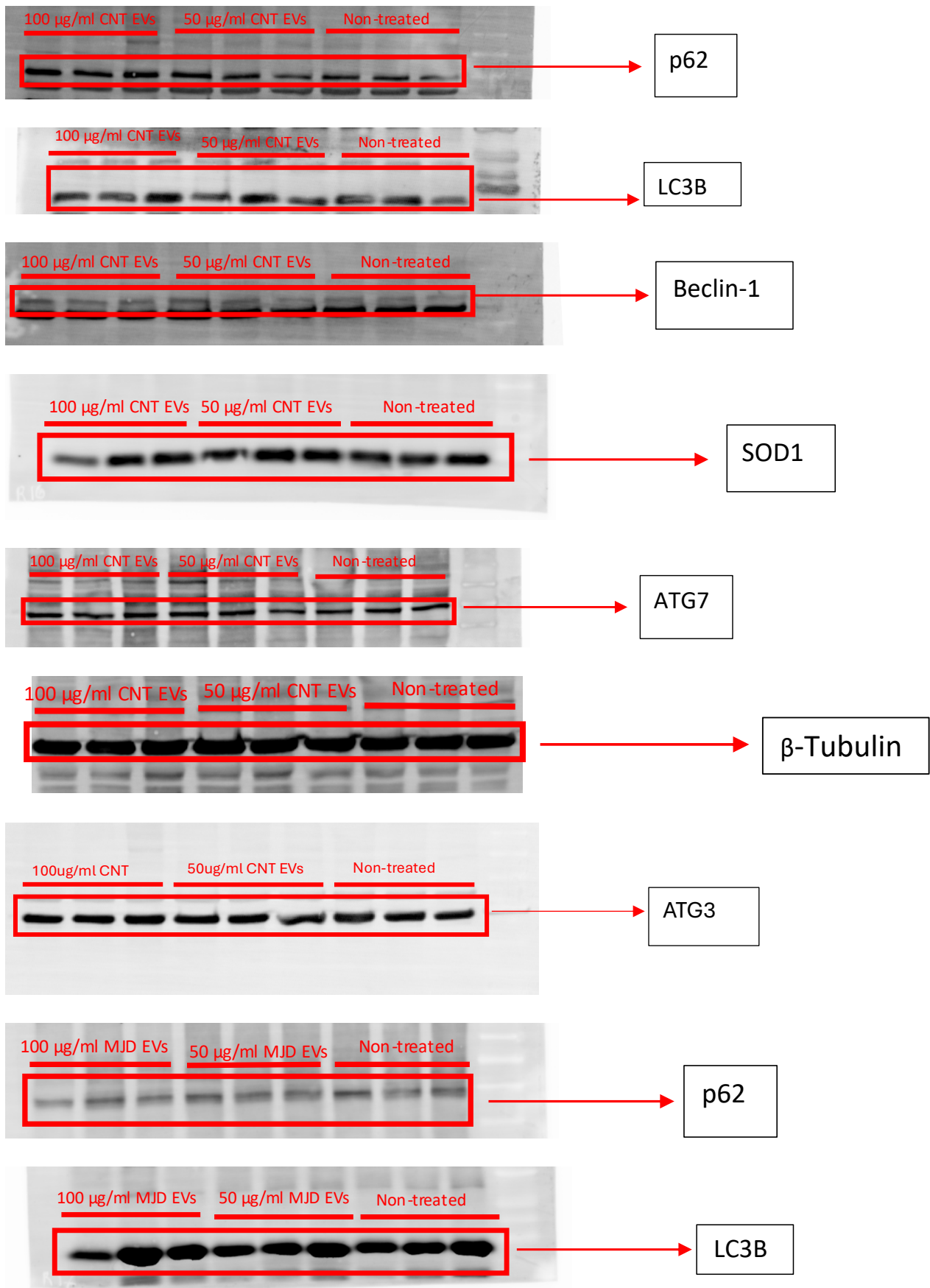

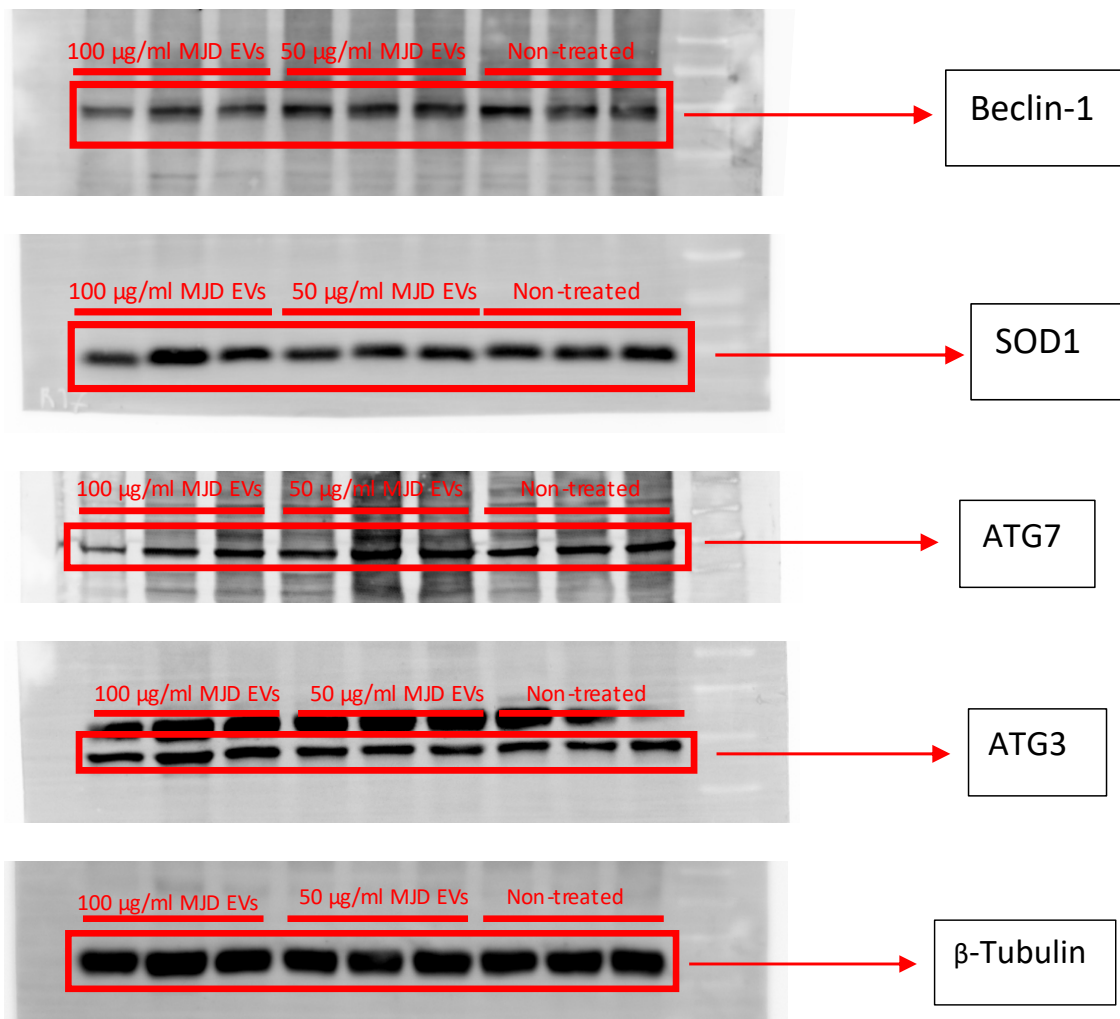

**Figure S2:**

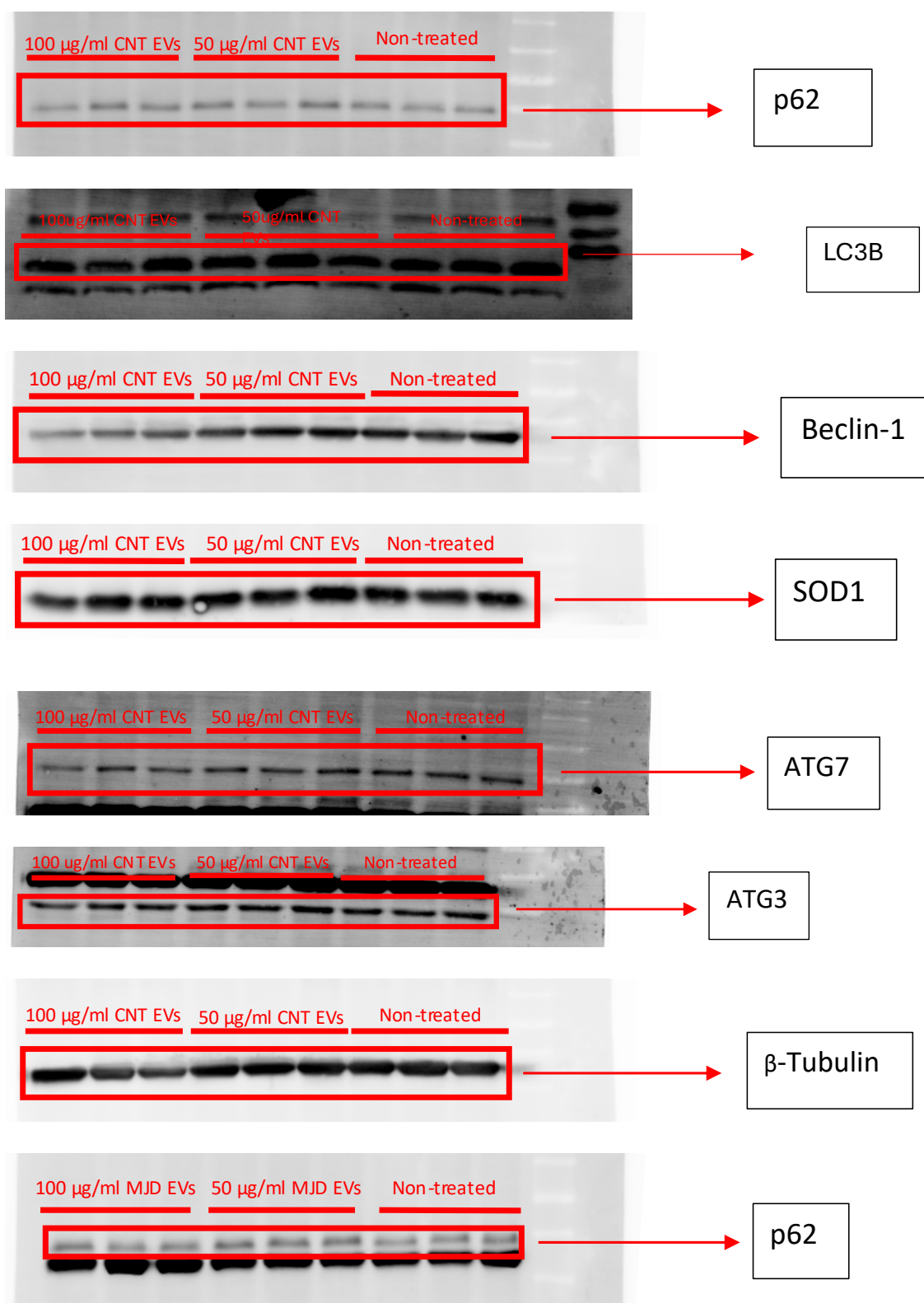

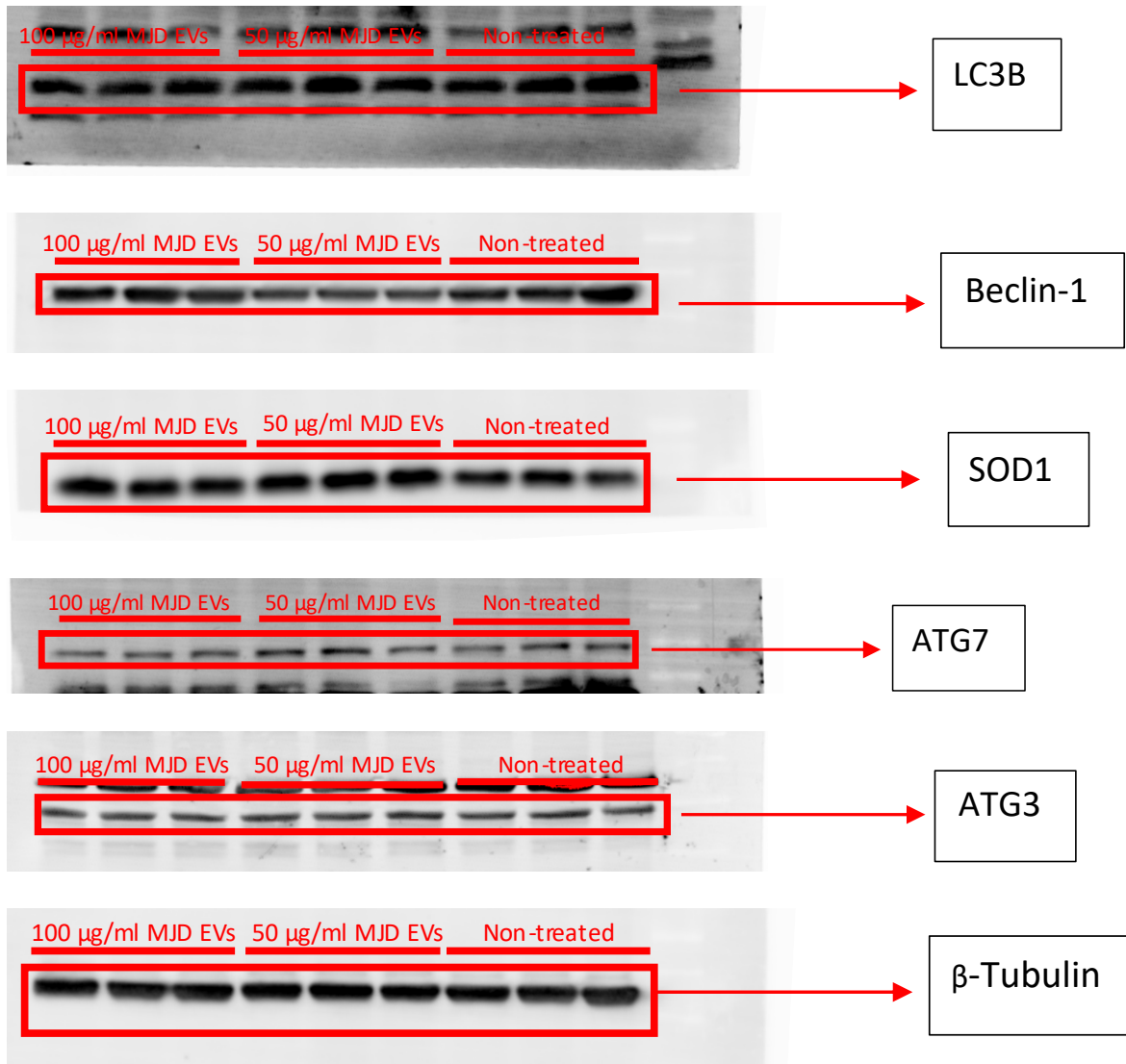

Supplement: Supplementary file 2 — Uncropped western blot membranes [file 41419_2025_7659_MOESM2_ESM.pdf]
